# Deep Neuro-Vision Embedded Architecture for Safety Assessment in Perceptive Advanced Driver Assistance Systems: The Pedestrian Tracking System Use-Case

**DOI:** 10.3389/fninf.2021.667008

**Published:** 2021-07-30

**Authors:** Francesco Rundo, Sabrina Conoci, Concetto Spampinato, Roberto Leotta, Francesca Trenta, Sebastiano Battiato

**Affiliations:** ^1^STMicroelectronics, ADG Central R&D Division, Catania, Italy; ^2^Department of Chemical, Biological, Pharmaceutical and Environmental Sciences, University of Messina, Messina, Italy; ^3^PerCeiVe Lab, University of Catania, DIEEI, Catania, Italy; ^4^IPLAB, Department of Mathematics and Computer Science, University of Catania, Catania, Italy

**Keywords:** driver drowsiness monitoring, deep learning, pedestrian tracking, adas, photoplethysmographic

## Abstract

In recent years, the automotive field has been changed by the accelerated rise of new technologies. Specifically, autonomous driving has revolutionized the car manufacturer's approach to design the advanced systems compliant to vehicle environments. As a result, there is a growing demand for the development of intelligent technology in order to make modern vehicles safer and smarter. The impact of such technologies has led to the development of the so-called Advanced Driver Assistance Systems (ADAS), suitable to maintain control of the vehicle in order to avoid potentially dangerous situations while driving. Several studies confirmed that an inadequate driver's physiological condition could compromise the ability to drive safely. For this reason, assessing the car driver's physiological status has become one of the primary targets of the automotive research and development. Although a large number of efforts has been made by researchers to design safety-assessment applications based on the detection of physiological signals, embedding them into a car environment represents a challenging task. These mentioned implications triggered the development of this study in which we proposed an innovative pipeline, that through a combined less invasive Neuro-Visual approach, is able to reconstruct the car driver's physiological status. Specifically, the proposed contribution refers to the sampling and processing of the driver PhotoPlethysmoGraphic (PPG) signal. A parallel enhanced low frame-rate motion magnification algorithm is used to reconstruct such features of the driver's PhotoPlethysmoGraphic (PPG) data when that signal is no longer available from the native embedded sensor platform. A parallel monitoring of the driver's blood pressure levels from the PPG signal as well as the driver's eyes dynamics completes the reconstruction of the driver's physiological status. The proposed pipeline has been tested in one of the major investigated automotive scenarios i.e., the detection and monitoring of pedestrians while driving (pedestrian tracking). The collected performance results confirmed the effectiveness of the proposed approach.

## 1. Introduction

Drowsiness is a symptom related to a lack of awareness which affects concentration, reaction time, and most seriously, safety (Schmidt, 1989; Rundo et al., 2019c). In the last few years, researchers discovered the mechanisms for which the level of attention is strongly correlated to the cardiac activity (Schmidt, 1989; Kurian et al., 2014; Rundo et al., 2019c).

The main functions of the heart are regulated by the Autonomic Nervous System (ANS). Specifically, the sympathetic and the parasympathetic nervous system, the two branches of ANS, are responsible for regulating many cardiac mechanisms, which are reflected in the attentional state of a subject (Schmidt, 1989). As stated previously, physiological signals represent a relevant data source to assess a subject's physiological condition (Kurian et al., 2014; Dastjerdi et al., 2017; Rundo et al., 2019d). The study of physiological signals have received much attention from the scientific community of the automotive industry (Kurian et al., 2014; Dastjerdi et al., 2017; Rundo et al., 2019d). Specifically, the growing proliferation of non-invasive medical devices to collect physiological parameters has led to the development of advanced new tools to be integrated into vehicle-environment. In this context, PhotoPlethysmoGraphic (PPG) signal has been proposed as a valid solution to analyze a subject's physiological status (Kurian et al., 2014; Dastjerdi et al., 2017; Rundo et al., 2019c,d). With the recent advances in safety awareness systems, the car manufacturers have spent a lot of efforts to develop innovative ADAS architectures based on PPG signal processing (Kurian et al., 2014; Rundo et al., 2019c). PPG is a convenient and simple physiological signal that provides information about the cardiac activity of a subject (Rundo et al., 2019d) and, therefore, the drowsiness status of a subject as well as pathologies which may indirectly have an impact on the subject's guidance^1^. In this work, we also focused on the use of the PPG signal for monitoring the subject's blood pressure. Several studies have pointed out that a robust driving risk assessment system leverages the so-called “driver fatigue condition” (which combines drowsiness and blood pressure monitoring) to perform a robust risk estimation (Husodo et al., 2018).

In this regard, the authors investigated promising solutions based on the use of a sensor framework to determine the level of driving safety through the driver's drowsiness assessment as well as the correlated blood pressure analysis (Littler et al., 1973; Husodo et al., 2018; Hui and Kan, 2019). Current solutions propose the use of wearable sensors or embedded devices equipped with such sensors in order to detect the first signs of fatigue^2^ (i.e., a significant and progressive lowering of the body temperature, the heart rate, etc.). Therefore, they require the use of invasive methodologies for the car driver that are often not feasible in automotive applications (Littler et al., 1973; Husodo et al., 2018; Hui and Kan, 2019).

Several reports have highlighted that cardiovascular diseases could have a major impact on the health of a subject, affecting also the level of attention (Wu et al., 2015). In order to gather information about users' health conditions (and specifically the “car driver” users), major interventions have been made for monitoring and analyzing the systolic and diastolic pressure of both healthy and hypertensive subjects through physiological signals. Preliminary studies have confirmed that there is a strong correlation between cardiovascular risk, drowsiness estimation, pressure level quantification, and physiological PPG signal (Littler et al., 1973; Schmidt, 1989; Kurian et al., 2014; Wu et al., 2015; Dastjerdi et al., 2017; Husodo et al., 2018; Hui and Kan, 2019; Rundo et al., 2019c,d). Inspired by the recent research studies, we defined the proposed work, providing an effective solution to overcome the limitations of the PPG acquisition. Due to the high sensibility of the PPG signal to Motion Artifacts (MA) generated with the body movements, *ad-hoc* sensing device combined with an innovative processing workflow was used (Dastjerdi et al., 2017; Rundo et al., 2019d). Therefore, it is not always easy to use methods based on PPG sampling and processing. In automotive field, the sensors of the PPG signal are usually embedded in the driver's steering in order to detect the needed physiological data (from the car driver hand placed on the steering) for drowsiness monitoring.

In Kurian et al. (2014) and Rundo et al. (2019c), the authors of these contribution have developed and patented an application that allows to detect the PPG waveforms of the driver from his/her hand placed in the car steering. Anyway, it is needed that the car-driver applies the hand in the PPG sensing devices arranged in the steering wheel; otherwise, the PPG signal cannot be collected (Kurian et al., 2014; Rundo et al., 2019c,d). Similarly, it would be advantageous to have a non-invasive system for monitoring the driver's blood pressure from PPG signal without having to wear such medical devices. In order to address the aforementioned issues, the authors propose an innovative pipeline for estimating some features of the PPG signal of any subject without the use of invasive devices. This paper presents an efficient Deep Learning pipeline designed to perform a non-invasive PPG features reconstruction using an innovative low frame-rate Motion Magnification algorithm (Wu et al., 2012). Moreover, we developed a proper Application System Framework (ASF) to assess both the driver drowsiness trough the usage of PPG signal features and the correlated blood pressure level, providing a robust evaluation of the driving safety.

The authors have a lot investigated the mentioned issue related to the car driver drowsiness monitoring as reported in Rundo et al. (2020a), Rundo et al. (2020b), Battiato et al. (2020), and Rundo et al. (2020c). They performed in-depth studies in relation to the robustness and performance of the delivered solutions. This paper is arranged into five sections. In section 2, the PPG based theory was introduced with a special focus to the physical principle that characterizes its formation. In section 3, we presented the used PPG sensing device. In section 4, the main scientific literature contributions are reported and discussed. In section 5, the whole proposed pipeline is described while in section 6 both the experiments and validation results are reported and discussed.

## 2. The PPG Signal: Underlying Physical Phenomena

PhotoPlethysmoGraphy is a non-invasive method for measuring blood flow in the cardiovascular circulatory system (Dastjerdi et al., 2017). More specifically, PPG is a physiological signal generally obtained through a combined optical-to-electric sensing system able to transduce the blood flow dynamic into electrical waveforms (Dastjerdi et al., 2017; Vinciguerra et al., 2019).

**Figure 1:**
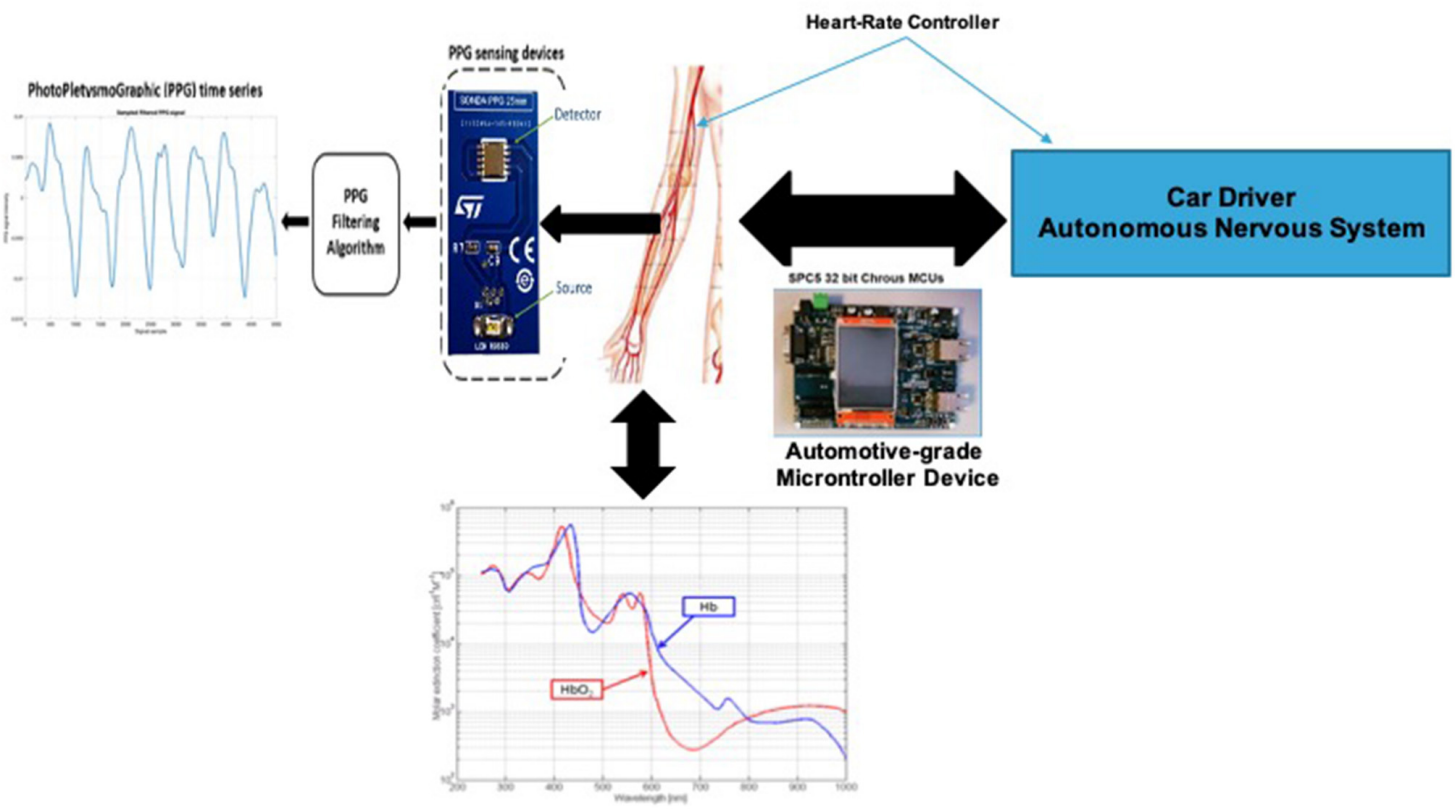
The PPG signal formation diagram.

Through the mentioned optical sampling methods, we are able to collect the PPG signal by illuminating the region of interest of the subject's skin with a Light Emitting Diode (LED). The used LED is coupled with a photo-sensing device that is able to capture the part of emitted light (photons), which is not absorbed by the subject blood flow (Back-Scattered signal; Vinciguerra et al., 2019). In particular, the photo-sensing device captures the part of reflected LEDs emitted light which is not absorbed mainly by the oxygenated hemoglobin (HbO2) and in residual form by the non-oxygenated (Hb) present in the subject's blood (Conoci et al., 2018; Vinciguerra et al., 2019). An electrical transduction circuit of the optical signal will complete the typical PPG signal acquisition pipeline. In Figure 1, the overall representation of the PPG pattern formation is reported. It shows the physical phenomenon underlying the formation of the PPG signal. Through the action of the ANS the heart rate of the subject is regulated (Schmidt, 1989). This regulation produces an impact in the arterial blood flow of the i.e., in the back-scattering data linked to the dynamics of HbO2 and Hb (Schmidt, 1989; Kurian et al., 2014; Rundo et al., 2019c,d). This collected data will be properly electrically transduced and processed by means of an automotive grade microprocessor, described in the next paragraphs.

## 3. The Proposed PPG Sensing System

Different hardware architectures have been proposed in order to sample the PPG signal (Agrò et al., 2014; Liu et al., 2016; Conoci et al., 2018; Vinciguerra et al., 2019). The authors proposed the use of an hardware architecture based on the employment of a photo-multiplier silicon device called SiPM (SiliconPhotoMultiplier) (Mazzillo et al., 2009, 2018; Liu et al., 2016; Vinciguerra et al., 2017; Rundo et al., 2018a).

**Figure 2:**
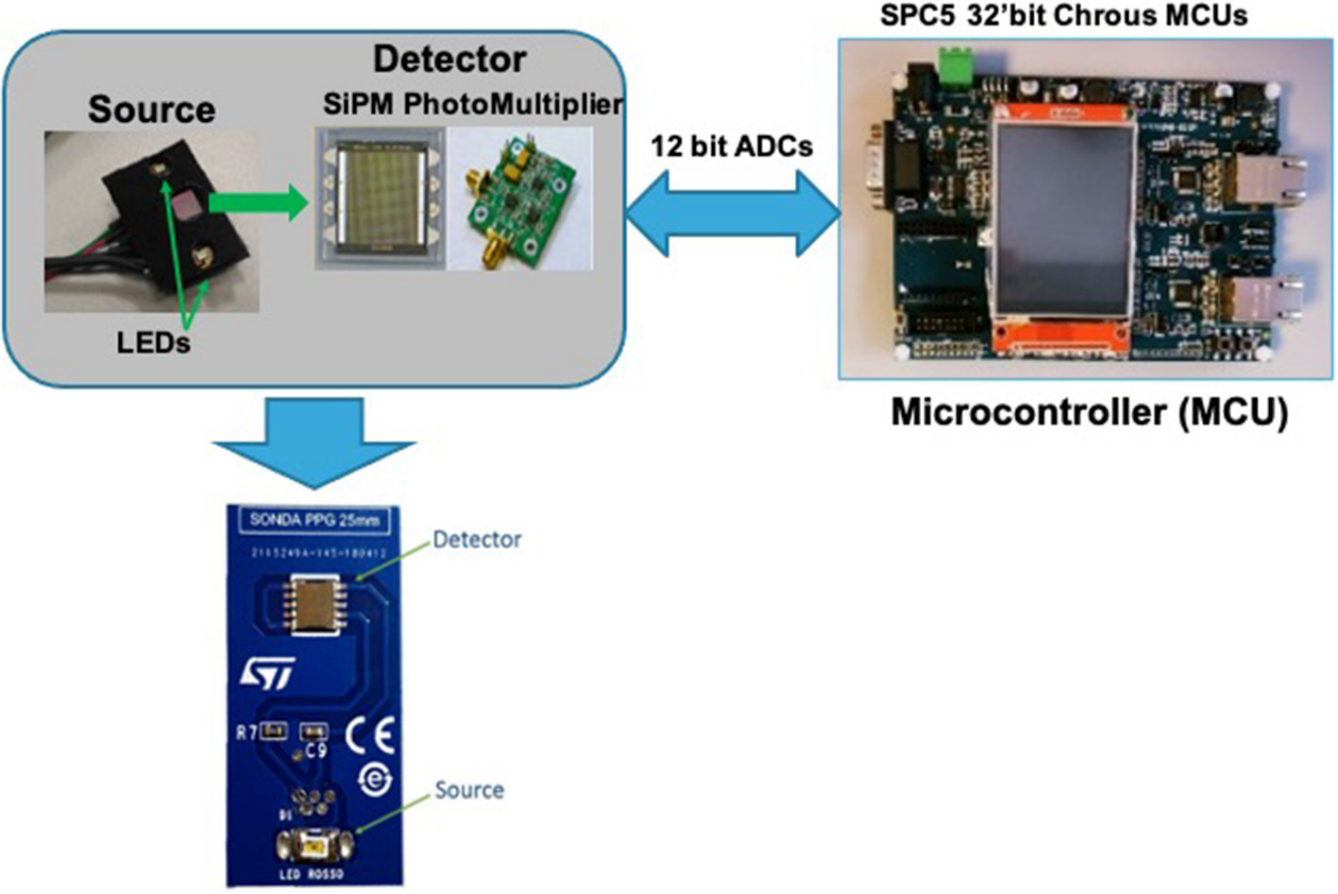
The proposed PPG Sensing Probe.

**Figure 3:**
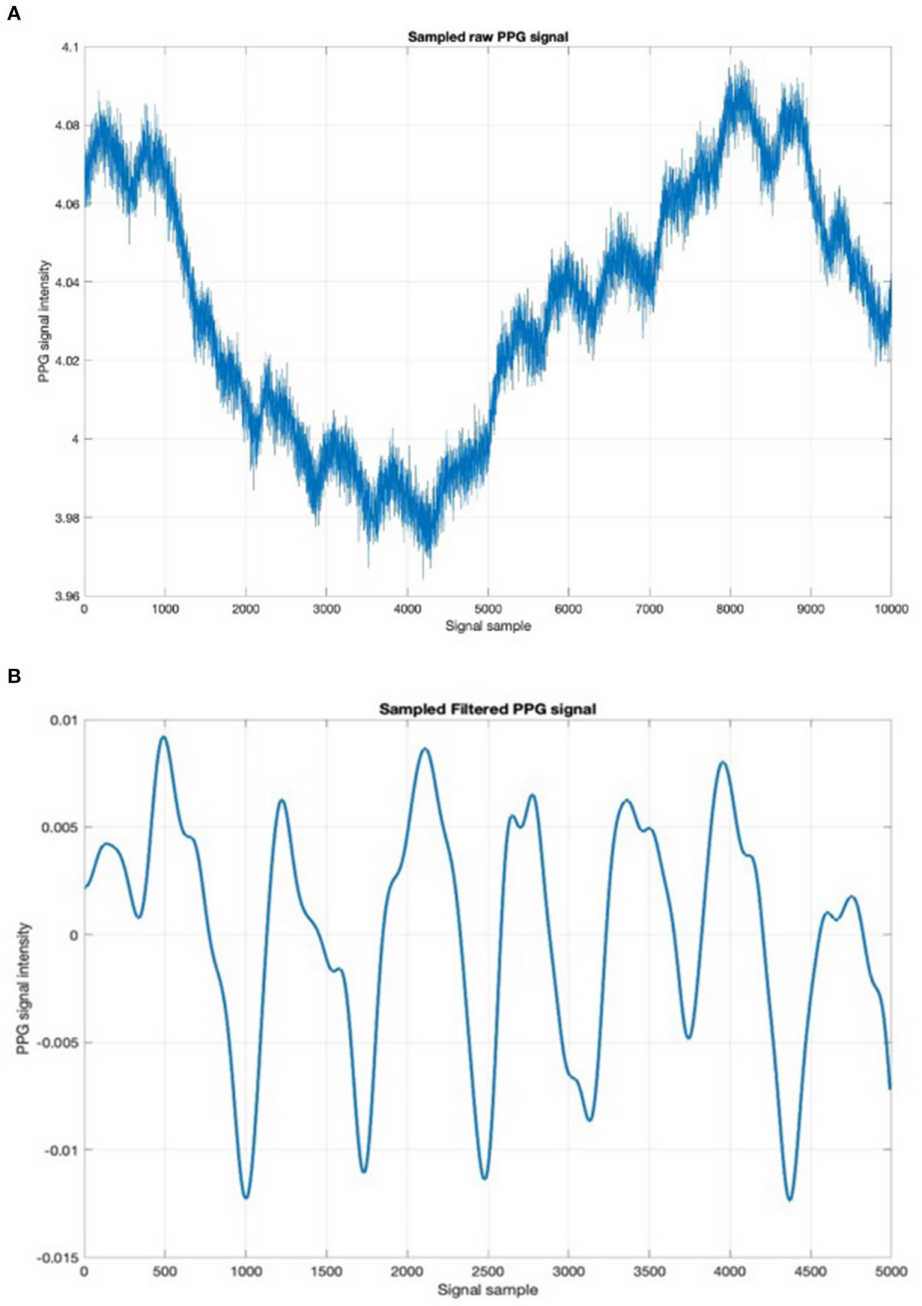
**(A)** Sampled raw PPG time-series. **(B)** Detail of the filtered PPG signal.

The designed sensing device is composed by two OSRAM LED emitters (SMD package) emitting at 850 nm. These leds are then used as optical light sources with a SiPM device (detector) which has a total area of 4.0 × 4.5 *mm*^2^ and 4,871 square microcells with 60 μm of the pitch. The proposed SiPM device has a geometrical fill factor of 67.4% and is packaged in a surface mount housing (SMD) of 5.1 × 5.1 *mm*^2^ total area. More details about the used hardware in Conoci et al. (2018), Agrò et al. (2014), Liu et al. (2016), and Mazzillo et al. (2009). Furthermore, to sample the PPG optical signal [through the embedded 12-bit Analogic to Digital Converter (ADC)] and to handle the implemented filtering and stabilization algorithms, the SPC5x 32-bit Chorus microcontroller was used (Vinciguerra et al., 2017; Mazzillo et al., 2018; Rundo et al., 2018a). Figure 2 shows the proposed designed PPG sensing hardware platform. The so sampled PPG raw signal comprises a pulsatile (“AC”) physiological signal, which is correlated to cardiac-synchronous changes in the blood volume, superimposed with a slowly varying (“DC”) component containing lower frequency sub-signals, which is correlated to respiration, thermoregulation, and so on (Vinciguerra et al., 2017; Mazzillo et al., 2018; Rundo et al., 2018a). Usually, the sampled raw PPG signal contains various types of noise: electronic, motion artifacts, micro-movements due to breathing and so on (Mazzillo et al., 2009, 2018; Liu et al., 2016; Vinciguerra et al., 2017; Rundo et al., 2018a). For this reason, it is needed to filter the raw PPG signal to obtain only the “AC” part of our interest (as it is directly correlated with the subject's heart activity) de-noised from the various kinds of noise. Figure 3A shows an instance of raw noised PPG signal sampled from the hand of a recruited subject. To achieve this purpose, a raw PPG signal filtering pipeline was implemented by authors. More in detail, in the aforementioned pipeline a Butterworth bandpass filter in the 0.5–10 Hz range was used. The authors also implemented an innovative, robust stabilization, and de-noising pipeline (motion artifacts, micro-breathing movements, etc.) called PPG-PRS (Choi et al., 2016; Rundo et al., 2018a, 2021). In Figure 3B, we reported a detail of the so filtered PPG waveforms. As shown in Figure 3B, a filtered steady-stable PPG signal is obtained by the deep pipeline proposed by the authors and called PPG-PRS (PPG Pattern Recognition System) (Choi et al., 2016; Rundo et al., 2018a, 2021). The so generated signal contains PPG waveforms which are compliant with the standard for this type of signal (Vinciguerra et al., 2017; Mazzillo et al., 2018).

The PPG-PRS technique is based on the use of a bio-inspired “reaction-diffusion” mathematical model that characterizes the two phases of cardiac activity (specifically it has been hypothesized that the diastolic phase is combined with a “reaction” dynamic and systolic to a phenomenon of “diffusion”). Through a cross-correlation analysis between the sampled PPG signal (filtered in the range 0.5–10 Hz) and the standard compliant PPG signal generated by the aforementioned reaction-diffusion mathematical model, the authors are able to recognize the waveforms of the PPG signal which are compliant with the standard and consequently discard those affected by noise or artifacts. Further implementation details of the PPG-PRS stabilization and de-noising pipeline can be found in Rundo et al. (2018a, 2021).

**Figure 4:**
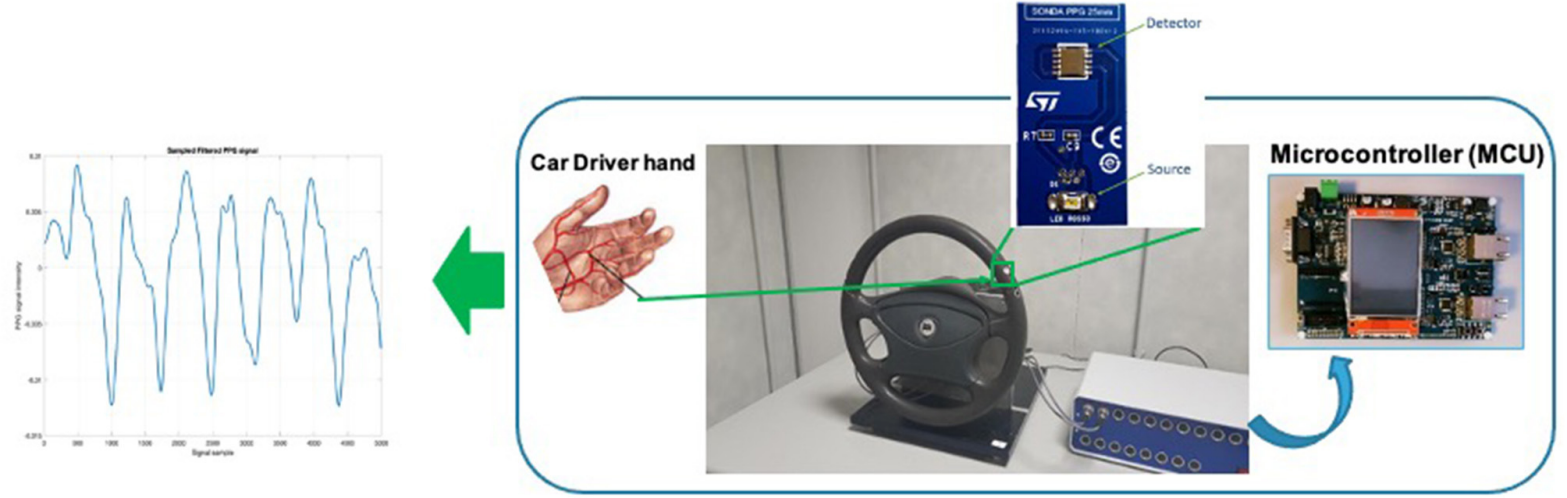
The overall scheme of the proposed PPG sensing device embedded in the car steering.

As introduced in the previous sections, in order to make a non-invasive sampling of the driver's PPG signal, the PPG sensing devices were embedded in the car steering in different positions, specifically, those where statistically the driver places the hands while driving. In this way, each time the driver also places a single hand in the steering part in which the PPG sensing device was embedded, the described signal formation mechanism will start working and the raw data will be sampled and then subsequently processed accordingly. In Figure 4, the overall scheme of the proposed PPG sampling pipeline is shown.

## 4. Related Works

Over the last decade, several researchers have investigated the issue that concern the evaluation of the driver's status with the physiological signals (Choi et al., 2016). Typically, these solutions are computationally expensive and invasive (Vavrinskỳ et al., 2010). Moreover, the continuous monitoring of physiological parameters during car driving requires high-performance systems (both hardware and software) to ensure high levels of safety while driving (Vavrinskỳ et al., 2010; Choi et al., 2016; Rundo et al., 2018a, 2021).

In this context, the PPG signal is the easiest to acquire among all the physiological signals that can be sampled by the driver (Vavrinskỳ et al., 2010; Choi et al., 2016). By simply allocating sensing devices (LED + SiPM) in the car steering wheel, the raw PPG signal of the driver can be easily sampled using the physical mechanism described in the previous paragraphs. The sampled PPG signal is post-processed by the microcontroller devices placed on the car (ADAS framework) to monitor drowsiness (Vavrinskỳ et al., 2010; Choi et al., 2016; Koh et al., 2017). In the other hand, to obtain the ElectroCardioGraphic (ECG) signal of the car driver, at least three contact-points would be required (Einthoven triangle; Rundo et al., 2018a). Therefore, the use of the PPG signal is preferred.

Another reliable source of data (which can be sampled in a non-invasive way) is the visual information referring to the driver's face and related facial expressions that seems to be correlated to the level of attention (Vural et al., 2007). For these reasons, many visio-based and physio-based solutions have been designed and implemented. In Koh et al. (2017), the authors analyzed the pattern of Heart Rate Variability (HRV) to monitor the drowsiness of the car driver. The HRV is a simple indicator suitable to measure the variation in the time interval between consecutive heartbeats (in milliseconds; Vavrinskỳ et al., 2010; Choi et al., 2016; Koh et al., 2017; Rundo et al., 2021). Several studies confirmed the correlation between a subject's attention level and the dynamic of related HRV, specifically with the analysis of such indicators extracted from the HRV called HF (High-Frequency power), LF (Low-Frequency power), and the LF/ HF ratio (Vural et al., 2007; Lee et al., 2011, 2019; Choi et al., 2017; Koh et al., 2017; Deng and Wu, 2019).

In Lee et al. (2019), a Convolutional Neural Network was used to classify drowsy/wakeful status. The CNN was fed by analyzing three types of recurrence plots (RPs) derived from the R-R intervals. The authors found that the simultaneous use of ECG and PPG signal (needed to collect the HRV) inevitably introduced a non negligible noise and artifacts. Another solution related to assess car-driver fatigue is reported in Choi et al. (2017) in which the authors proposed a system to measure the car driver's emotional and physiological status coming from a wearable device placed on the wrist. As well as previous work, a pre-processing pipeline was developed in order to extract such valid time-series from the acquired signals. The so extracted features were classified by using a Support Vector Machine method. The results confirmed the effectiveness of the proposed solution in detecting and distinguishing the driver's physiological status. In Lee et al. (2011), the researchers proposed an innovative method to detect driver drowsiness combining Computer Vision and Image Processing approaches. Specifically, they evaluated the PPG signal waveform in order to record changes from awake to a drowsy state. At this stage, they detect the eye region through the use of template matching in combination with a Genetic algorithm to analyze the eye's behavior. In order to derive the final classification, PPG drowsy signals are evaluated with eye motion behavior to provide more robust and effective results. Deng and Wu (2019) proposed a novel approach, called DriCare, to estimate driver drowsiness by using a face-tracking algorithm. The authors designed a new method to individuate 68 key points in facial regions with the aim of evaluating drivers' fatigue status. Another promising work that investigates the problem of assessing car driver's fatigue state is Jabbar et al. (2020) in which the authors developed a Convolutional Neural Networks to classify drowsiness status. Specifically, the authors used facial landmarks as input data for their proposed model. The main contribution of their work is the development of a deep learning-based system that can be easily integrated into a car environment. As introduced in this scientific contribution, several studies investigated the driving safety assessment, in addition to the detection of the level of car driver attention as well as to monitor driver blood pressure. In the scientific literature many approaches deals with the arterial stiffness or the estimation of blood pressure with the advantage of Deep Learning (DL) methods and PPG signal based analysis. In Monte-Moreno (2011), the authors used a photoplethysmography sensor in order to estimate the diastolic and systolic blood pressure in a non-invasive way. In particular, the PPG waveform was used to gather the features used as input data in a various machine learning algorithm to be able to estimate the systolic (SBP) and diastolic (DBP) blood pressure and the blood glucose level (BGL). The results confirmed that Random Forest achieved better prediction estimation confirming the relationship between the shape of PPG waveform and blood pressure level. The effectiveness of Machine Learning (ML)-based techniques have been furtherly confirmed by experimental results that showed the relationship there is among the PPG waveform, blood pressure and glucose levels. Slapničar et al. (2019) investigated the problem of detecting Blood Pressure (BP) using an ML-based architecture. The authors fed a novel spectro-temporal Deep Neural Network (DNN) with the PPG and its first and second derivative to be able to overcome limitations that concern the cuff-based devices. The ability to compute dependency between PPG waveforms and blood pressure and the effectiveness of the proposed model was confirmed by means of leave-one subject-out experiments. In Alty et al. (2007), a pipeline to predict arterial stiffness (an indicator correlated to subject blood pressure) was proposed by the authors. Moreover, with the purpose of examining cardiovascular disease they performed classification of subjects into high and low aortic pulse wave velocity (PWV) classes. The collected results confirmed the effectiveness of the proposed Support Vector Machine (SVM) based solution. In Rundo et al. (2018b), a novel approach was described by the authors in order to estimate cardiovascular disease risk by means blood pressure. The method reported in Rundo et al. (2018b) measures the subject blood pressure by analyzing the correlated PPG signal. In Huynh et al. (2018), the authors used the averaging Impedance Plethysmography (IPG) for the detection of Pulse Transit Time (PTT) in order to estimate the blood pressure. The tests showed that the estimation of blood pressure (BP) achieved interesting results (RMSE: 8.47 ± 0.91 and 5.02 ± 0.73 mmHg for systolic and diastolic levels, respectively). On the other hand, the previous approaches needs the use of invasive medical devices and sometimes require the need to sample the ECG signal in addition to the PPG and therefore impracticable in the automotive application.

## 5. Methods and Materials

### 5.1. The Driving Safety Assessment Through Physiological Driver Analysis

Recent studies have highlighted the need to assess the car driver's physiological state in order to create highly safety automotive-grade applications. As previously reported, the PPG is a less-invasive signal suitable to provide useful information about the physiological condition of the driver. In fact, the main limitation of the existing solutions consists in the integration of sensors inside the vehicle to acquire the physiological signals. Most of the solutions propose a PPG signal sampling methodology based on the usage of such sensors placed on the car steering wheel. However, this implies that the driver has to maintain an unnatural behavior i.e., the hands constantly over the sensors. Moreover, if these sensors no longer work while driving, the classic pipelines would not be able to collect the signal, representing a serious risk during the real-time safety assessment (Kurian et al., 2014; Rundo et al., 2018b, 2021). In Figure 4, we schematized the PPG sampling pipeline embedding the sensors placed on the steering wheel.

With this regard, we designed an innovative pipeline which combines an enhanced version of the Motion Magnification analysis and Deep Learning approach for a non-invasive processing of the car driver PPG signal. As a result, the proposed approach is able to overcome the aforementioned critical issues. More specifically, we developed an innovative module called Vision2PPG Reconstruction Pipeline that reconstructs the features of the driver's PPG signal from a visual data. Through the use of a video-camera placed on the car dashboard, we recorded the driver's face, tracking the facial movements over the frames. Finally, by *ad-hoc* processing of these visual data we developed *ad-hoc* algorithm to reconstruct such features of the car driver's PPG signal even in absence of native sensing data. The use of Deep Architectures properly trained will complete the proposed pipeline.

### 5.2. The Vision2PPG Reconstruction Pipeline

**Figure 5:**
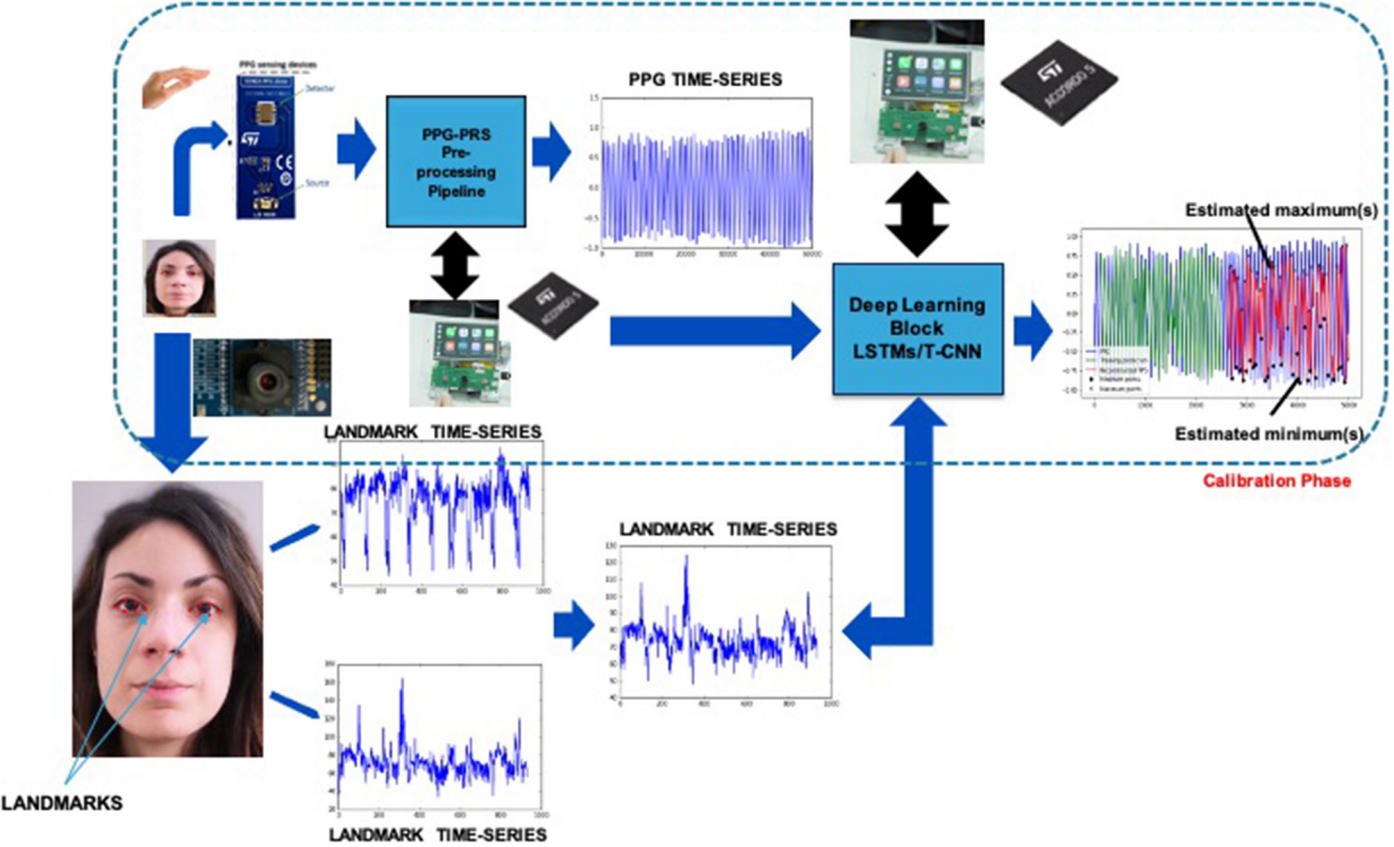
The overall scheme of the proposed Vison2PPG Reconstruction pipeline.

In this section, we go through the details of the developed Vision2PPG pipeline. The proposed pipeline consists of a PPG sensing device designed to perform a preliminary system calibration and a camera device that captures the dynamic of the driver's frontal face. The collected data have been used to extract facial descriptors (landmarks). The designed module is based on Computer Vision techniques which cover specific requirements regarding the automotive certification ASIL-x (Dastjerdi et al., 2017; Vinciguerra et al., 2019). A deep learning algorithm (embedded in an ASIL-B microcontroller as firmware) completes the proposed pipeline by correlating the subject's face descriptors with the corresponding PPG waveforms. The overall flowchart of the proposed Vision2PPG pipeline is shown in Figure 5. A detailed description of the pipeline will be reported in the following paragraphs. As PPG sensors, we implemented the sensing system design described in the previous section. The designed four PPG sensing probes will be embedded in the driver's steering wheel equidistant from each other. The designed LEDs will emit at the wavelength of 850 nm (near infrared). The first phase of the proposed pipeline is the training-calibration task. The training-calibration phase includes a learning stage in which the pipeline determines the correlation between visual time-dynamic of the segmented face descriptors (landmarks) with the associated PPG features. This module was designed to collect enough data to characterize the car driver drowsiness as well as the correlated blood pressure level. At this stage, the raw PPG signal is firstly sampled by using the designed coupled LED-SiPM sensing probes (Mazzillo et al., 2018; Rundo et al., 2018a). We also applied the PPG-PRS (means PPG Pattern Recognition System) algorithm to the so collected raw PPG signal in order to obtain a compliant filtered PPG time-series (Rundo et al., 2018a, 2021).

**Figure 6:**
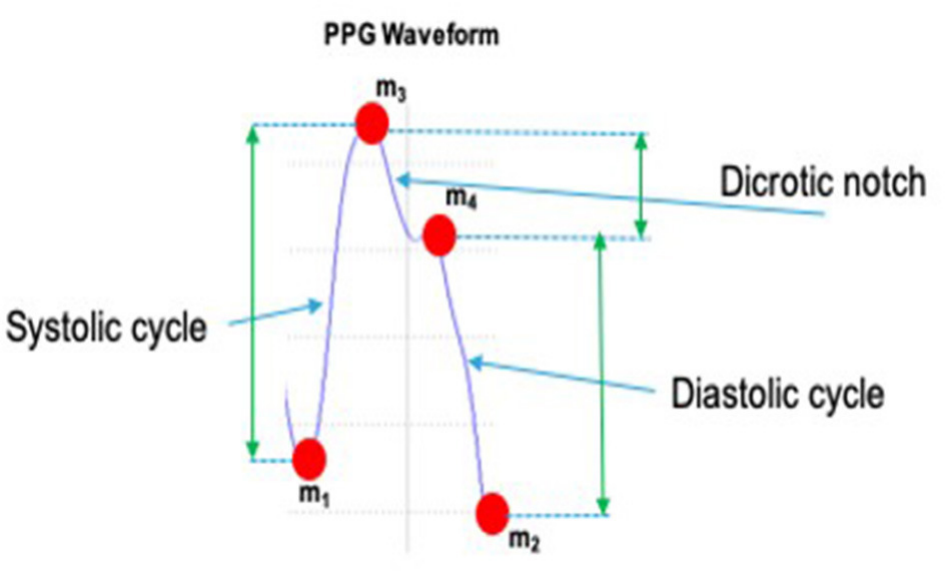
A filtered compliant PPG waveform with a detail of such extreme points m1, m2, m3, m4.

**Figure 7:**
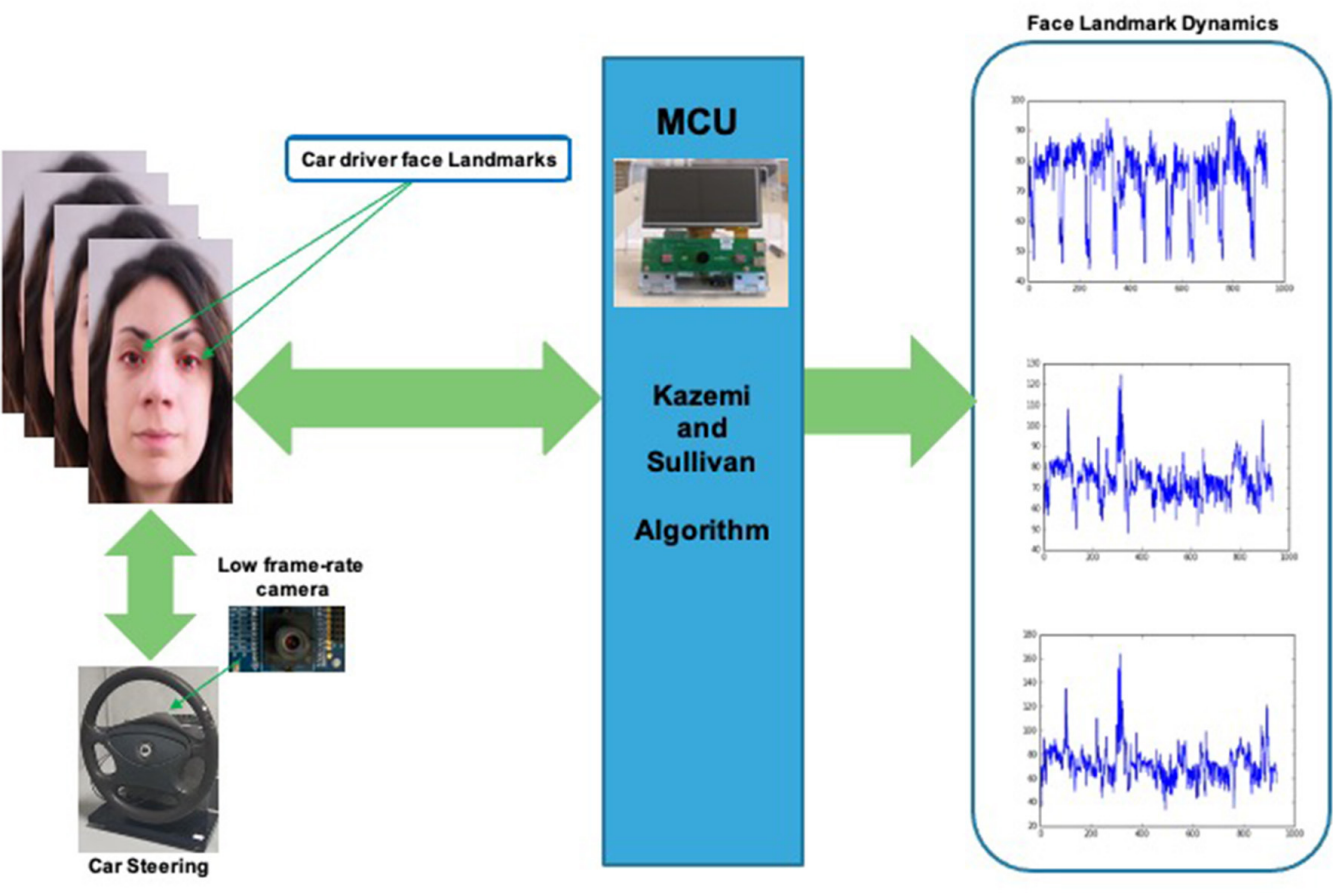
The proposed enhanced Motion Magnification pipeline.

The PPG-PRS preliminary filters the raw PPG signal cutting off artifacts and noise through the use of a Butterworth bandpass filter in the range 0.5–10 Hz. Moreover, a study of the first and second derivatives of the filtered PPG signal was implemented in the PPG-PRS algorithm. This analysis allows to detect the minimum and maximum extreme points of each selected compliant PPG waveform. Finally, by means of a second order dynamic and Reaction-Diffusion system, which emulates the physiological phenomenon of the PPG signal formation, the PPG-PRS algorithm is able to stabilize the sampled PPG signal. The configuration of the PPG-PRS algorithm is exactly the one reported in the scientific contribution (Rundo et al., 2018a). In Figure 6, we depicted an instance of a filtered PPG compliant waveform, identifying the corresponding extreme points *m*_1_, *m*_2_, *m*_3_, *m*_4_. The second part of the proposed Vision2Physio reconstruction pipeline is composed by the enhanced motion magnification module. More in detail, we performed the PPG sampling simultaneously with the recording of a video sequence of the subject's frontal face by using a low frame-rate camera device under normal light conditions. Specifically, we used a device with a max resolution of 2.3 Mpx and 50 fps as framerate. Several studies have demonstrated that the face of a subject performs visual micro-movements closely related to the cardiac pumping activity (systolic and diastolic phase; Oh et al., 2018). The PPG signal (as a cardiac-related signal) is strongly correlated to the aforementioned micro-movements (Balakrishnan et al., 2013). In order to make these micro-movements visible at naked eyes, some authors have designed innovative motion magnification techniques which require a video-camera devices with high frame-rate (on average, frame rate ranging from 10 Kfps up; Rubinstein et al., 2013). Motion Magnification refers to amplifying facial micro-movements in order to reveal the flow of blood (Balakrishnan et al., 2013; Rubinstein et al., 2013; Oh et al., 2018). However, the method originally proposed for motion magnification showed an evident issue in relation to automotive applications as it requires, as mentioned, the need for a high frame-rate video device (of the order of Kfps). This constraint is not easily covered in automotive field mainly for reasons of costs and sustainability of the underlying hardware. For this reason, by extending a previous version already implemented, the authors propose in this contribution a different motion magnification method which addresses the mentioned issues. The preliminar version of the proposed enhanced motion magnification algorithm was reported by the authors in Trenta et al. (2019). With this regard, we firstly developed a method to process video frames depicting face sequences of the car driver in order to preliminary identify significant landmarks or descriptors on the subject's face. The proposed pipeline is shown in Figure 7.

In order to detect the aforementioned landmarks, the approach implemented in Kazemi and Sullivan (2014) was used. This algorithm locates the (*x, y*) coordinates of 68 facial points (landmarks) describing the facial structures (i.e., mouth, eyes, nose, etc.) on a subject's face image. The approach is based on the usage of a pre-trained learning model (Kazemi and Sullivan, 2014). The main advantage of this algorithm consists in obtaining near real-time high-quality landmarks recognition and tracking even with a low frame-rates video. More details in Kazemi and Sullivan (2014). In our previous solution reported in Trenta et al. (2019). both the hardware PPG signal sensing system and the landmarks detection algorithm were quite different. The PPG sensing system used green LEDs. Furthermore, although Kazami and Sullivan's algorithm was used to retrieve the landmarks of the driver's face, the number of used landmarks were significantly lower than the total number (68 descriptors), specifically, there were only two (landmarks adjacent to the eyes). For the reconstruction of the PPG features from the visual subject's face frames, a deep classifier based on LSTM was used. However, this model only reconstructed the minimum points of each PPG waveform. The setup reported in Trenta et al. (2019) allowed us to prove that the motion magnification model we proposed was able to achieve optimal performance in the automotive field. However, the pipeline described in Trenta et al. (2019) was implemented in a prototype system by National Instruments that required data buffering and therefore the overall system response time was significantly slowed down. Furthermore, considering that the pipeline used only two car driver landmarks adjacent to the eyes, in certain scenarios this visual data could be no longer available (for instance a scenario in which the driver wearing sunglasses) and therefore the pipeline would be no longer applicable. These limitations have been largely overcome in the motion magnification pipeline herein proposed. Formally, we have defined the reconstruction of landmarks dynamics by means of Kazemi and Sullivan based function ψ_*KS*_(.). If we indicate with *I*_*t*_*k*__(*x, y*) the captured *M* × *N* gray-level (or luminance gray-level channel in case of color camera device) video frame of the car's driver at the instant *t*_*k*_, the *i*−*th* dynamic landmark ℓ_*i*_(*t*_*k*_, *x*_*l*_, *y*_*l*_) is reconstructed as follow:

(1) $$\begin{array}{l} \ell_{i}\left({{t_{k},x}_{l}}_{i},y_{l_{i}}\right)=\psi_{KS}\left(I_{t_{k}}\left(x,y\right)\right);\;k=1..N_{f};i=1..N_{L} \end{array}$$

where ℓ_*i*_(*t*_*k*_, *x*_*li*_, *y*_*li*_) represents the pixel intensity variation of the i-th landmark identified at the space position (*x*_*li*_, *y*_*li*_) on the frame while *N*_*f*_ represents the number of captured frames and *N*_*L*_ represents the number of identified landmarks (i.e., 68 as per Kazemi et al. based algorithm). Therefore, in the proposed pipeline the whole set of landmarks was used making the proposed method more robust than the version reported in Trenta et al. (2019). We did a test in a scenario where the driver is wearing sunglasses. While the Trenta et al. (2019) method is no longer applicable, our approach continues to work by presenting overlapping performances (see experimental results session for more details). Furthermore, it must be said that the Trenta et al. (2019) method is not applicable to the reconstruction of the driver's pressure level (see next paragraphs) due to the reduced number of landmarks. Moreover, the usage of a PPG sensing device emitting at near-infrared spectrum allowed to have a native PPG signal more detailed than that obtained using green light (as in Trenta et al., 2019) and that is for some specific characteristics implicit in the physiological process of the signal formation (Schmidt, 1989; Kurian et al., 2014; Dastjerdi et al., 2017; Conoci et al., 2018; Rundo et al., 2019d; Vinciguerra et al., 2019).

Once the representative landmark dynamics of the driver have been identified, we proceed analysing the so collected descriptors time-series ℓ_*i*_(*t*_*k*_, *x*_*li*_, *y*_*li*_) in order to correlate their intensity temporal dynamics with the underlying cardiac activity. The proposed method does not require high frame-rate camera devices as for the method to which it is inspired (Littler et al., 1973). It requires a normal commercial vision device having a framerate in the range ≥40 fps. To model the aforementioned relationship between face landmarks and the cardiac activity of the analyzed driver, the authors propose two deep learning frameworks: one based on the usage of Deep Long Short-Term Memory (D-LSTM) architectures and the other one based on the usage of 1D Temporal Deep Dilated Convolution Neural Network (1D-TDCNN) network. Moreover, in order to improve the robustness of the proposed safety assessment pipeline, we introduce a classical Deep Convolutional Neural Network (D-CNN) for car driver's eyes tracking to be correlated with level of attention or drowsiness. In the following sections, some details of the proposed framework will be outlined.

### 5.3. The Deep Learning Framework

As described in the previous section, the proposed Deep Learning framework consisted of two parts: (i) a deep architecture (D-LSTM or 1D-TDCNN) employed to classify driver's PPG signal or in case it is no longer available, the driver landmarks dynamics ℓ_*i*_(*t*_*k*_, *x*_*li*_, *y*_*li*_) retrieved from the Vision2PPG Reconstruction pipeline, (ii) a Depp CNN used to perform eyes-based visual classification of the car-driver drowsiness. In the following subsections, the authors proceed to the scientific description of each block of the proposed Deep Learning pipeline.

#### 5.3.1. The Car Driver Landmarks Deep Classifier

**Figure 8:**
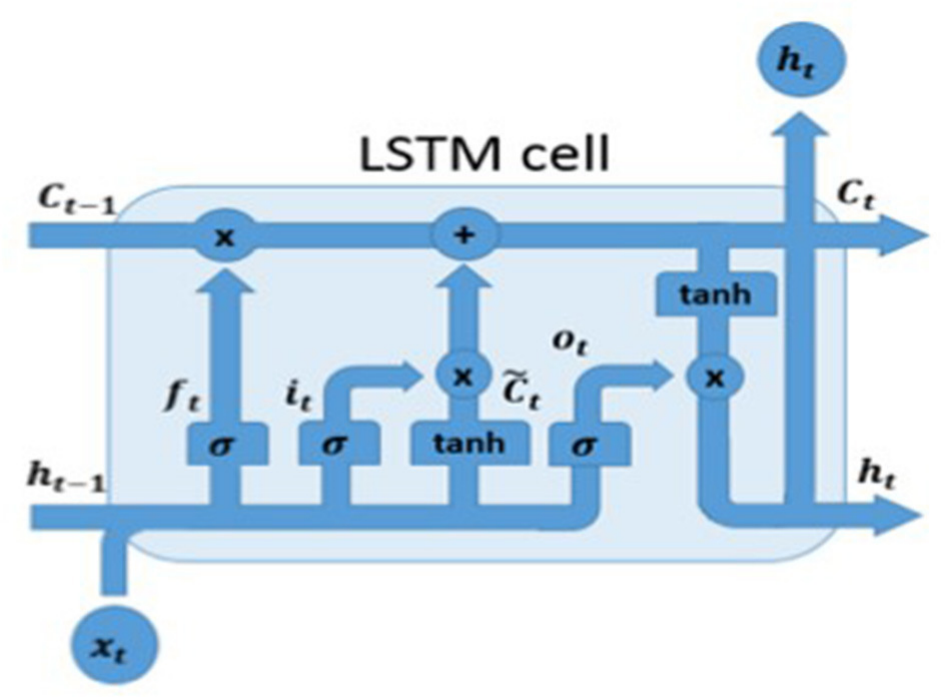
A prototype of the LSTM cell.

As introduced, we propose two deep learning basic solutions to correctly classify the driver visual landmarks and correlate them with such PPG features (specifically: the extreme points of the PPG signal). The first setup uses *ad-hoc* Deep Long Short-Term Memory (D-LSTM) framework. In particular, our D-LSTM network is based on Vanilla architecture, firstly proposed by Hochreiter and Schmidhuber (1997). As mentioned, the authors have already used such LSTM-based architecture to address similar automotive application with respect to what it is herein described (Trenta et al., 2019). Moreover, vanilla D-LSTM architectures have been largely employed in the automotive field (Monte-Moreno, 2011; Koh et al., 2017; Vinciguerra et al., 2017). We remark that in this contribution, the authors significantly improved the approach previously described in Trenta et al. (2019). Specifically, the proposed approach allows to better reconstruct the PPG signal (more extreme points) and consequently improves the performance in terms of drowsiness classification. Furthermore, the proposed approach allows to estimate the corresponding level of blood pressure. In the next paragraphs, more details about the performance and benchmarking of the proposed approach. Let's introduce the designed deep platform. The proposed D-LSTM architecture is composed of one input layer, two hidden layers and one output layer. In order to significantly improve the classification performance, the input layer was designed with 64 input units and, consequently, the two hidden layers with 64 and 128 cells, respectively. Finally, the output layer, that consists in 1 cell, will provide the predicted PPG samples that will be used to determine the extreme points *m*_1_, *m*_2_, *m*_3_, *m*_4_. In Figure 8, we reported the basic unit structure of the used D-LSTM. The mathematical model which represents the learning model of the cell is reported as follow:

(2) $$f_{t}=\;\sigma (W_{f}•\left[h_{t-1},\;x_{t}\right]+\;b_{f}$$

(3) $$i_{t}=\;\sigma (W_{i}•[h_{t-1},\;x_{t}]+\;b_{i}$$

(4) $$\begin{array}{l} {\tilde{C}}_{t}=\tanh\left(W_{C}•\left[h_{t-1},x_{t}\right]+\;b_{C}\right) \end{array}$$

(5) $$\begin{array}{l} C_{t}=\;f_{t}*C_{t-1}+\;i_{t}*\;{\tilde{C}}_{t} \end{array}$$

(6) $$o_{t}=\;\sigma (W_{o}•[h_{t-1},\;x_{t}]+\;b_{o}$$

(7) $$\begin{array}{l} h_{t}=o_{t}*\tanh\left(C_{t}\right) \end{array}$$

From Figure 8, it is clear that given *x*_*t*_ as input vector, the previous output cell *h*_*t*−1_ as well as the previous cell memory *C*_*t*−1_, the current cell output *h*_*t*_ and the current cell memory *C*_*t*_, are suitable to determine what information the D-LSTM has to be stored. Equations (4)–(7) allows to model the behavior of the proposed D-LSTM. In order to improve the overall performance, a batch normalization and dropout layer have been added to the output of each LSTM layer. Our model was trained with an initial learning rate of 10^−3^, a batch size set to 512 and with the maximum number of training epochs set to 200. During the training-calibration phase of each recruited driver subject, the suggested Deep LSTM is able to learn the correlation there is among the selected facial landmark time-series ℓ_*i*_(*t*_*k*_, *x*_*li*_, *y*_*li*_) and the corresponding sampled PPG signal. The designed Deep LSTM pipeline will produce in output the predicted PPG waveforms from which the extreme points *m*_1_, *m*_2_, *m*_3_, *m*_4_ will be detected as reported in Figure 6. After several tests we found that not all the identified landmarks are correlated with the subject's cardiac activity and then in order to obtain acceptable performances, in terms of reconstruction of the PPG signal features, only a subset of landmarks can be processed. Furthermore, thanks to the implemented D-LSTM architecture we have verified that a more simple composite landmarks signal can be used for addressing the needed PPG reconstruction task. Specifically, we perform the following computation:

(8) $$\begin{array}{l} \mu_{\ell }\left(t_{k}\right)=\frac{1}{N_{L}}\overset{N_{L}}{\underset{j=1}{\sum }}\ell_{j}\left({{t_{k},x}_{l}}_{j},y_{l_{j}}\right)\;\forall \;t_{k} \end{array}$$

**Figure 9:**
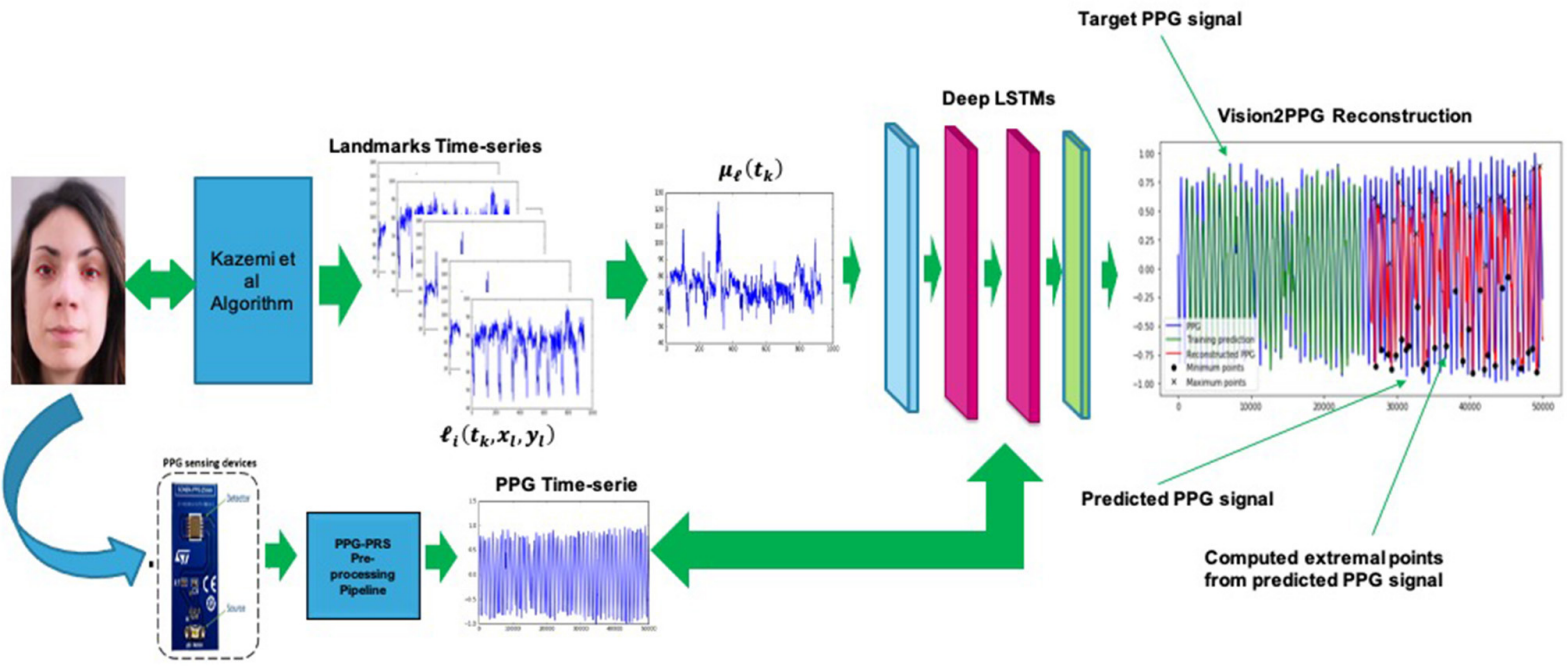
The proposed D-LSTM pipeline for PPG features estimation. The corresponding extreme points are highlighted with black circle and “x” symbol in the predicted PPG waveforms (red signal).

The so computed (properly normalized) signal μ_ℓ_(*t*_*k*_) will be given as input of the designed D-LSTM. Consequently, the architecture will be trained to find the correlation between the input signal μ_ℓ_(*t*_*k*_) and the corresponding PPG signal. Figure 9 shows the overall scheme of the proposed D-LSTM. Basically, the extreme points *m*_1_, *m*_2_, *m*_3_, *m*_4_ for each predicted PPG waveforms (see Figure 6 for more details) are computed by performing the same analysis applied by the mentioned PPG-PRS algorithm (Rundo et al., 2018b) i.e., by analyzing the first and second derivative of the D-LSTM estimated PPG signal.

**Figure 10:**
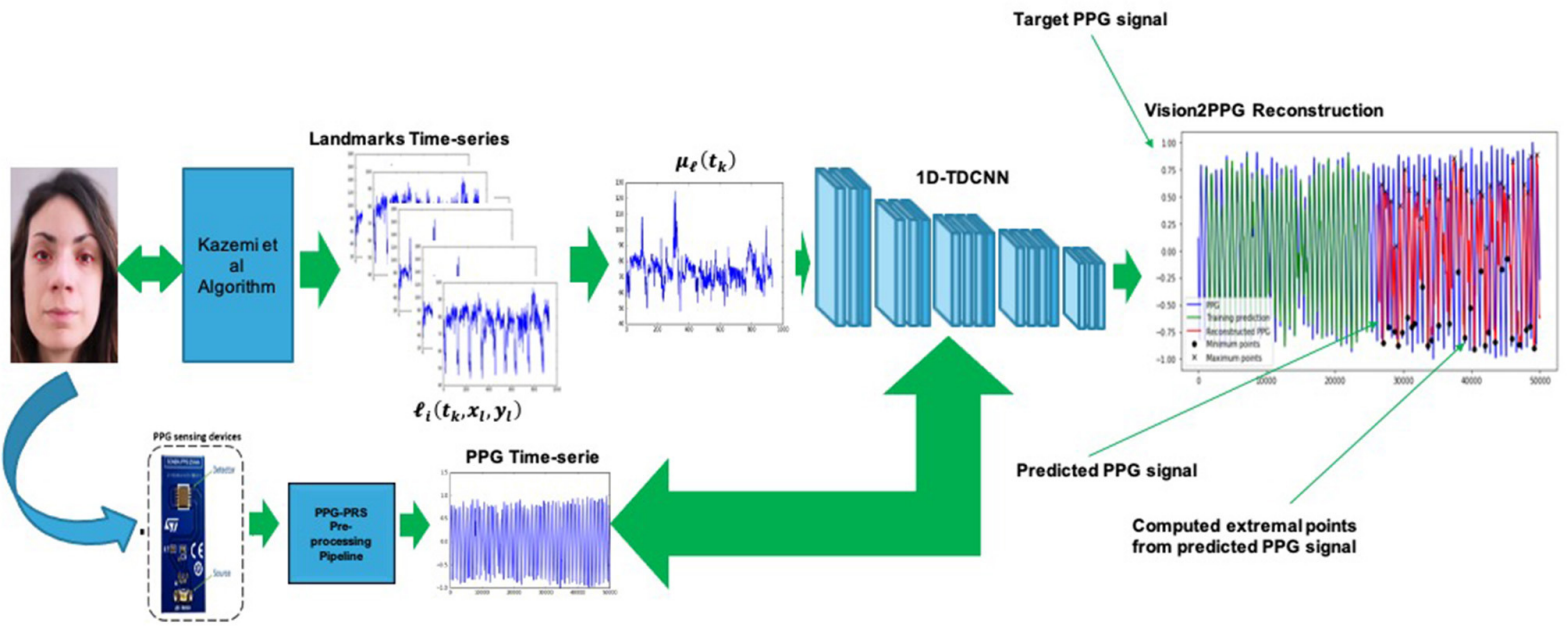
The proposed 1D-TDCNN pipeline for PPG features estimation. The corresponding extreme points are highlighted with black circle and “x” symbol in the predicted PPG waveforms (red signal).

The second analyzed deep architecture that we have implemented to correlate the visual features and the corresponding PPG signal, is based on the use of a temporal deep architecture. Specifically, *ad-hoc* 1D Temporal Deep dilated Convolutional Neural Network (1D-TDCNN) has been developed (Zhao et al., 2019). The main building block consists of a dilated causal convolution layer that operates over the time steps of each sequence (Zhao et al., 2019). The proposed 1D-TDCNN includes multiple residual blocks, each containing two sets of dilated causal convolution layers with the same dilation factor, followed by normalization, ReLU activation, and spatial dropout layers. Furthermore, a 1 × 1 convolution is applied to adapt the number of channels between the input and output. Specifically, we implemented a 1D-TDCNN composed of 25 blocks with a downstream SoftMax layer. Each of the deep blocks comprise a dilated convolution layer with 3 × 3 kernel filters, a spatial dropout layer, another dilated convolution layer, a ReLU layer, and a final spatial dropout. The dilation factor size starts of a factor equal to 2 and it will be increased for each block till the value of 16. As for LSTM based solutions, the so-designed 1D-TDCNN output represents predicted extreme points of the PPG signal. The following Figure 10 shows the proposed 1D-TDCNN based solution.

To validate the effectiveness of the proposed Deep pipelines in reconstructing such features of the original PPG signal, we computed the Fast Fourier Transform (FFT) of a signal obtained through the difference between the PPG reconstructed minimum points (Trenta et al., 2019). As described in the introductory part of this contribution, the HRV monitoring is one of the classic structured physiological method for determining the drowsiness level of a subject. As widely confirmed in scientific literature, the HRV can be computed performing a proper Fast Fourier Transform (FFT) of a differential physiological signal obtained from ECG i.e., by means of the distance R-Peak to R-Peak as well as from PPG through the distance of the minimum points of two consecutives waveforms (Lee et al., 2011; Rundo et al., 2018b; Trenta et al., 2019). Therefore, a well robust measurement of HRV of a subject (car driver in our application) can be obtained by computing the FFT spectrum of the PPG based physiological differential signal. In case of the PPG signal is unavailable the predicted PPG minimum points reconstructed by Vision2PPG block will be used. Once the HRV is computed, by means of classical analysis based on the usage of the HF (High-Frequency power), LF (Low-Frequency power) and the LF/ HF ratio, the drowsiness of a subject is easily detected and monitored (Lee et al., 2011, 2019; Choi et al., 2017; Deng and Wu, 2019). More details about the performance validation of the proposed deep pipelines will be reported in the experimental results section.

As soon as the proposed Deep architecture -both D-LSTM either 1D-TDCNN- has learned the correlation there is among the driver's facial landmarks and extreme points of the relative PPG signal, the training-calibration phase will be dropped, and therefore the system will work feed-forward. The calibration phase of the whole described pipeline requires a 15/20 s of PPG signal (the proposed sensing device is able to execute an acquisition at 1 KHz) with the relative visual frames (acquired at 50 fps) and it will be needed to perform only at once. Obviously, this system will be enabled by the driver hardware control unit whenever the physiological signal of the driver will not be detected for some reasons by the PPG sensors embedded in the car's steering. Therefore, compared to the previous approach proposed by the authors in the contributions (Rundo et al., 2018a,b, 2019d; Trenta et al., 2019), the method herein described allows to reconstruct more discriminating PPG features (the four extremal points of the PPG waveform against only the minimums of the previous version). Furthermore, the Vision2PPG pipeline with the downstream classifier is much more robust than in scenarios where some visual landmarks are no longer available. By using a 1D TDCNN, the long-range temporal dependencies of the PPG signal are better treated allowing an effective changes detection in the state of attention induced by the autonomic nervous system of the driver. In addition, the proposed pipeline provides features that allow to also obtain a less invasive and cuff-less assessment of the driver's pressure level not possible in the solutions previously proposed in Trenta et al. (2019).

#### 5.3.2. The Car Driver Eye's Tracking Trough Deep CNN Pipeline

**Figure 11:**
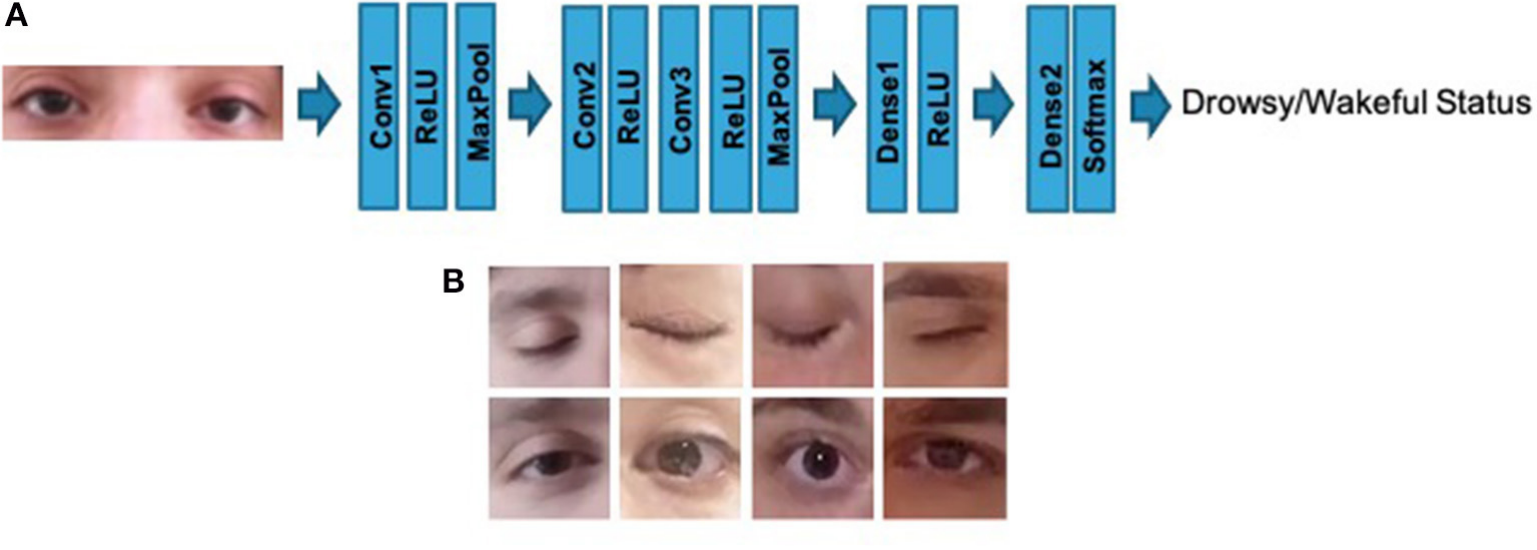
**(A)** The proposed D-CNN architecture. **(B)** Some instances of D-CNN input visual data.

**Figure 12:**
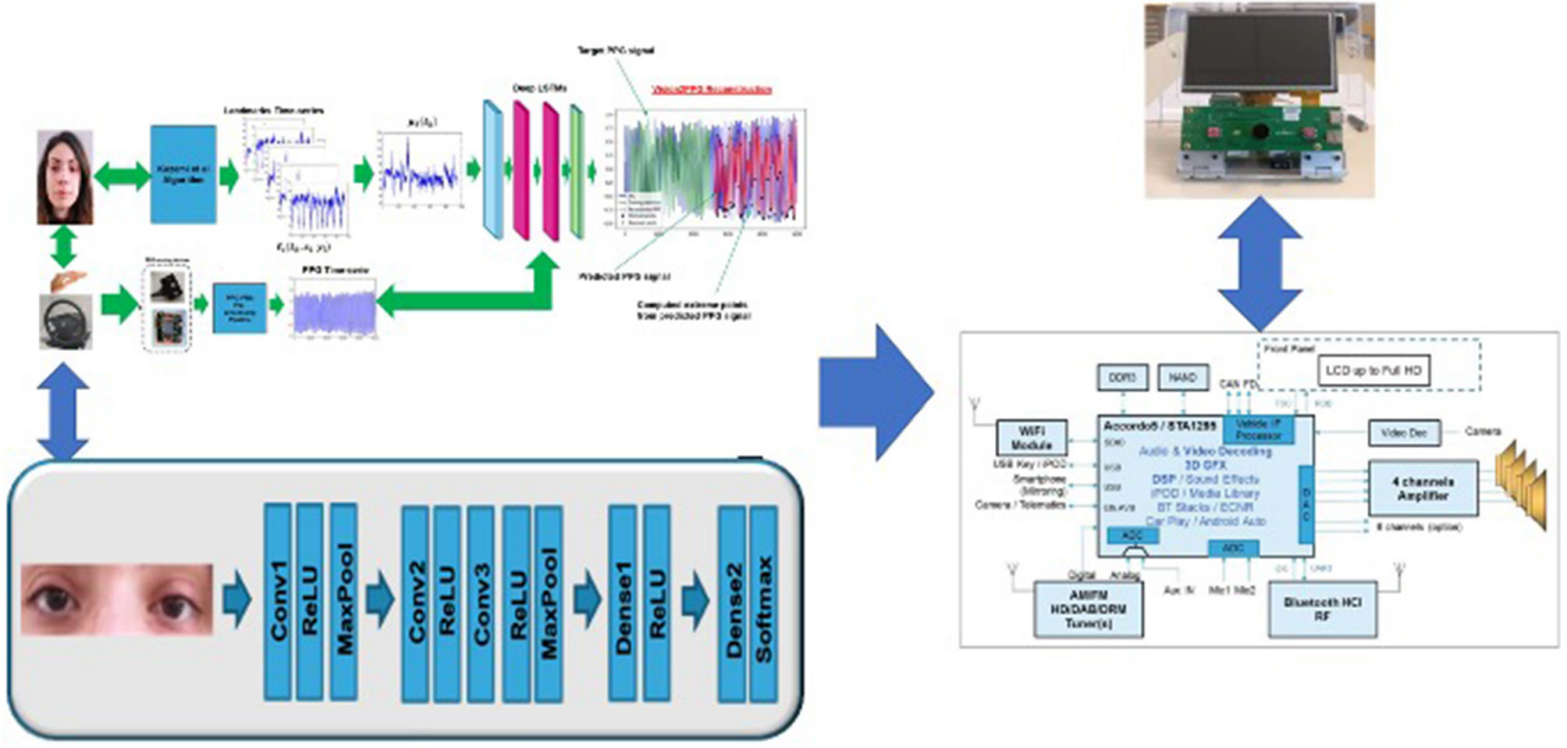
The STA1295A Accordo5 platform hosting the D-LSTM Vison2PPG Reconstruction pipeline as well as D-CNN architecture.

In order to have a simultaneous safety monitoring pipeline without technology overlap with the previously described blocks, the authors implemented a further assisted pipeline based on the usage of *ad-hoc* Deep Convolutional Neural Network (D-CNN). The proposed 2D D-CNN network is quite simple and includes three convolutional layers each of which has a ReLU activation layer (with batch-normalization), such 2 × 2 max pooling layers except for the last convolutional layer. The first convolutional layer performs 32 operations with 3 × 3 kernel filters, where the second and the third shows 64 and 128 kernel filters of 3 × 3, respectively. A stack of densely connected layers and Softmax complete the proposed D-CNN pipeline in order to perform two classes classification of the input visual data i.e., drowsy/wakeful status of the analyzed driver. Fine-tuning is done for 100 epochs using Adam optimizer and cross entropy as loss function. To carry out the experiments, we also set the learning rate to 0.001 and the batch size to 32. In Figure 11, the proposed D-CNN architecture. The input visual frame of the D-CNN is a patch depicting a single eye (77 × 77 resolution), which we segment from the driver's face using the algorithm proposed in Viola and Jones (2001). This algorithm does not require an annotated images dataset to work and can it be easily carried on embedded hardware architectures while maintaining remarkable performance both in segmentation and in execution speed. More details in Viola and Jones (2001). The proposed full D-CNN has been designed in order to get it portable to such embedded hardware solution ASIL-B certified i.e., STA1295 Accordo5 MCU^3^ as described in this work. Specifically, the D-CNN (as well as the previous D-LSTM/1D-TDCNN pipelines) will be hosted in the STA1295 Accordo5 embedded automotive grade DUAL ARM A7 which includes a 3D-GFX accelerator cell suitable for this kind of processing (Rundo et al., 2019a). In Figure 12, we reported a scheme of the overall platform. All input data (PPG samples and visual frames) will be properly stored in such buffers allocated in the EMI memory of the STA1295 MCU platform.

### 5.4. The Car-Driver Blood Pressure Estimation Pipeline

The application described in this section regards the monitoring of the driver blood pressure as strongly correlated to the driving safety and driver attention level. We propose a novel solution for measuring blood pressure from such features of the PPG signal as well as from the corresponding facial video frames of the subject by using the proposed Vision2PPG reconstruction pipeline (in case the PPG signal unavailability). We remark that our proposed blood pressure estimation pipeline works both in the scenario in which the PPG is unavailable and in the case in which the physiological signal was correctly sampled by the sensing probe. As described, by means of the Vision2PPG pipeline, we will obtain the extreme points of the subject's PPG signal. From these predicted data, by means of a suitably configured Shallow Neural Network (Rundo et al., 2019c), the authors are able to discriminate normal blood pressure subjects from those who had pressure values beyond the norm. The blood pressure estimation pipeline has been designed in order to be ported as firmware running in the STA1295 Accordo5 embedded automotive processor. Once the set of characteristic extreme points of the PPG waveforms have been collected (by means of the Vision2PPG reconstruction pipeline or from native PPG signal), we are able to characterize the subject's cardiac activity (systole, dicrotic, and diastole phases) on which the level depends the blood pressure (Rundo et al., 2018b). Let's formalize this application. In Figure 6, we have reported an instance of compliant PPG waveform with highlighted extreme points *m*_*x*_. For each pair of PPG waveforms *PPG*^*j*^, *PPG*^*j*+1^ we define the following indicators:

(9) $$\begin{array}{l} \varphi =[m_{1}^{j},m_{2}^{j},\;m_{3}^{j},m_{4}^{j},dx_{i}^{j},dy_{i}^{j},mAI^{J}]\forall \;j=1..(N^{PPG}-1)\\ \;;i=1,2,3,4 \end{array}$$

(10) $$m_{i}^{j}=(x_{m_{i}^{j}},y_{m_{i}^{j}})\;,\forall \;j=1..(N^{PPG}-1)\;;i=1,2,3,4$$

(11) $$dx_{i}^{j}=x_{m_{i}^{j+1}}-x_{m_{i}^{j}},\;\forall \;j=1..(N^{PPG}-1)\;;i=1,2,3,4$$

(12) $$dy_{i}^{j}=y_{m_{i}^{j+1}}-y_{m_{i}^{j}}\;\forall \;j=1..(N^{PPG}-1)\;;i=1,2,3,4$$

(13) $$mAI^{j}=((y_{m_{3}^{j}}-y_{m_{1}^{j}})-y_{m_{4}^{j}})/(y_{m_{3}^{j}}-y_{m_{1}^{j}})$$

where *mAI*^*J*^ is a modified version of the so-called Augmentation Index, usually computed for measuring the arterial stiffness (Vavrinskỳ et al., 2010) while *N*^*PPG*^ represents the number of estimated PPG waveforms. The other indicators reported in the Equations (9)–(12) are able to characterize cardiac cycles and, therefore, the relative blood pressure level. The input of the above-mentioned Shallow Neural Network (SNN) is represented by the elements of the vector φ. The SNN is a Fully Connected Multi-Layers Network with two hidden layers of 500 and 300 neurons and a binary output. It was designed with the target of learning the correlation there is among the so computed input elements in φ and the associated diastolic and systolic blood pressure values. Furthermore, the aforementioned SNN was trained with the Scaled Conjugate Gradient backpropagation (SCG) algorithm (Rundo et al., 2019c). As output, the SNN framework will produce a value that may be treated like a discriminating flag able to differentiate the subject showing normal pressure values (0) with respect to the hypertensive or hypotensive subject (1). Specifically, we calibrate the system in order to detect if the subject has a pressure level that is on average 15% higher or lower than a reference value. The set 120/80, which indicates 120 mmHg for systolic pressure and 80 mmHg for diastolic pressure, has been considered normal blood pressure values (as confirmed by the team of physiologists who assisted us in this study). On the other hand, higher or significantly lower (15%) values are considered anomalous. It should be noted that the proposed system is able to monitor and discriminate even different pressure levels (with respect to the classic 120/80 mmHg) requiring a different and adequate calibration. This is to cover the cases of moderate hypotension/hypertension affected subjects who physiologically present a normal blood pressure level a little different from 120/80 mmHg. For these subjects, the pipeline calibration phase will refer to different reference pressure values. In any case, during the calibration phase of the Vision2PPG recognition system, the training of the SNN block will be performed in order to correlate the blood pressure (reference value and current value) and PPG levels of the subject preparing to drive. The proposed pipeline has been tested and validated in one of the most interesting automotive scenario: the pedestrian tracking system.

### 5.5. The Deep Network With Criss-Cross Attention for Pedestrian Tracking System

The tracking of pedestrians while driving is certainly one of the most important aspects in the field of safety automotive requirements. The detection and subsequent monitoring of pedestrians in the driving scene allows the automatic driver assistance system to continuously validate if the driving dynamics and the level of attention are compatible with the presence of pedestrians in the scene. Many authors have investigated this relevant issue by analyzing the advantages inherent in the use of deep learning architectures (Tian et al., 2015; Song et al., 2018; Jeon et al., 2019; Bhola et al., 2020). The authors investigated several interesting object detection and tracking architecture backbones to be adapted to pedestrian tracking. Specifically, the researchers found that deep learning systems embedding attention mechanisms significantly increase the performance in classification and segmentation of the underlying backbones. For these reasons, we found it useful to implement an innovative network that included the recent self-attention context through the use of Criss-Cross layers (Huang et al., 2019). More in detail, for each source image/feature pixel, an innovative Criss-Cross attention module computes the contextual information of all the correlated pixels on its Criss-Cross path. This attention pre-processing combined with further recurrent operations allow the Criss-Cross method to leverage the full-image dependencies during the learning session of the deep network (Huang et al., 2019). Let us formalize the attention processing embedded in the Criss-Cross layer. Given a local feature map *H* ∈ *R*^*C*×*W*×*H*^ where *C* is the original number of channels while *W* × *H* represents the spatial size of the generated feature map trough a Deep Convolutional Network. The Criss-Cross layer applies two preliminary 1 × 1 convolutional layers on *H* in order to generate two feature maps *F*_1_ and *F*_2_, which belong to *R*^*C*′×*W*×*H*^ and in which *C'* represents the reduced number of channels due to dimension reduction with respect to original (C). Let define an *Affinity* function suitable to generate the Attention-Map $A_{M}\in R^{\left(H+W-1\right)\times \left(W\times H\right)}$. The affinity operation is so defined. For each position *u* in the spatial dimension of *F*_1_, we extract a vector $F_{1,u}\in R^{C}$. Similarly, we define the set $\Omega_{u}\in R^{\left(H+W-1\right)\times C}$ by extracting feature vectors from *F*_2_ at the same position *u*, so that, $\Omega_{i,u}\in R^{C^{\prime }}$ is the i-th element of Ω_*u*_. Taking into account the above operations, we can define the introduced Affinity operation as follows:

(14) $$\begin{array}{l} \delta_{i,u}^{A}=F_{1,u}\Omega_{i,u}^{T} \end{array}$$

where $\delta_{i,u}^{A}\in D$ is the affinity potential i.e. the degree of correlation between features *F*_1,*u*_ and Ω_*i,u*_, for each i = [1,…,H + W - 1], and *D* ∈ *R*^(*H*+*W*−1)×(*W* × *H*)^. Then, we apply a softmax layer on *D* over the channel dimension to calculate the attention map *A*_*M*_. Finally, another convolutional layer with 1 × 1 kernel will be applied on the feature map *H* to generate the re-mapped feature ϑ ∈ *R*^*C*×*W*×*H*^ to be used for spatial adaptation. At each position *u* in the spatial dimension of ϑ, we can define a vector $\vartheta_{u}\in R^{C}$ and a set $\Phi_{u}\in R^{\left(H+W-1\right)\times C}$. The set Φ_*u*_ is a collection of feature vectors in ϑ having the same row or column with position *u*. At the end, the final contextual information will be obtained by an *Aggregation* operation defined as follows:

(15) $$\begin{array}{l} H_{u}^{\prime }=\overset{H+W-1}{\underset{i=0}{\sum }}A_{M}^{i,u}\Phi_{i,u}+H_{u} \end{array}$$

where $H_{u}^{\prime }$ is a feature vector in *H*′ ∈ *R*^*C*×*W*×*H*^ at position *u* while $A_{M}^{i,u}$ is a scalar value at channel *i* and position *u* in *A*_*M*_. The so defined contextual information $H_{u}^{\prime }$ is then added to the given local feature *H* to augment the pixel-wise representation and aggregating context information according to the spatial attention map *A*_*M*_. These feature representations achieve mutual gains and are more robust for semantic segmentation. Anyway, the criss-cross attention module is able to capture contextual information in horizontal and vertical directions but the connections between one pixel and its around is not processed. To overcome this issue, the authors firstly introduced the Criss-Cross methodology proposed a Recurrent Criss-Cross processing in which classic Criss-Cross operations can be unrolled into *R* loops. We defined *R* = 2 for our purpose as suggested by the original description (Huang et al., 2019).

**Figure 13:**
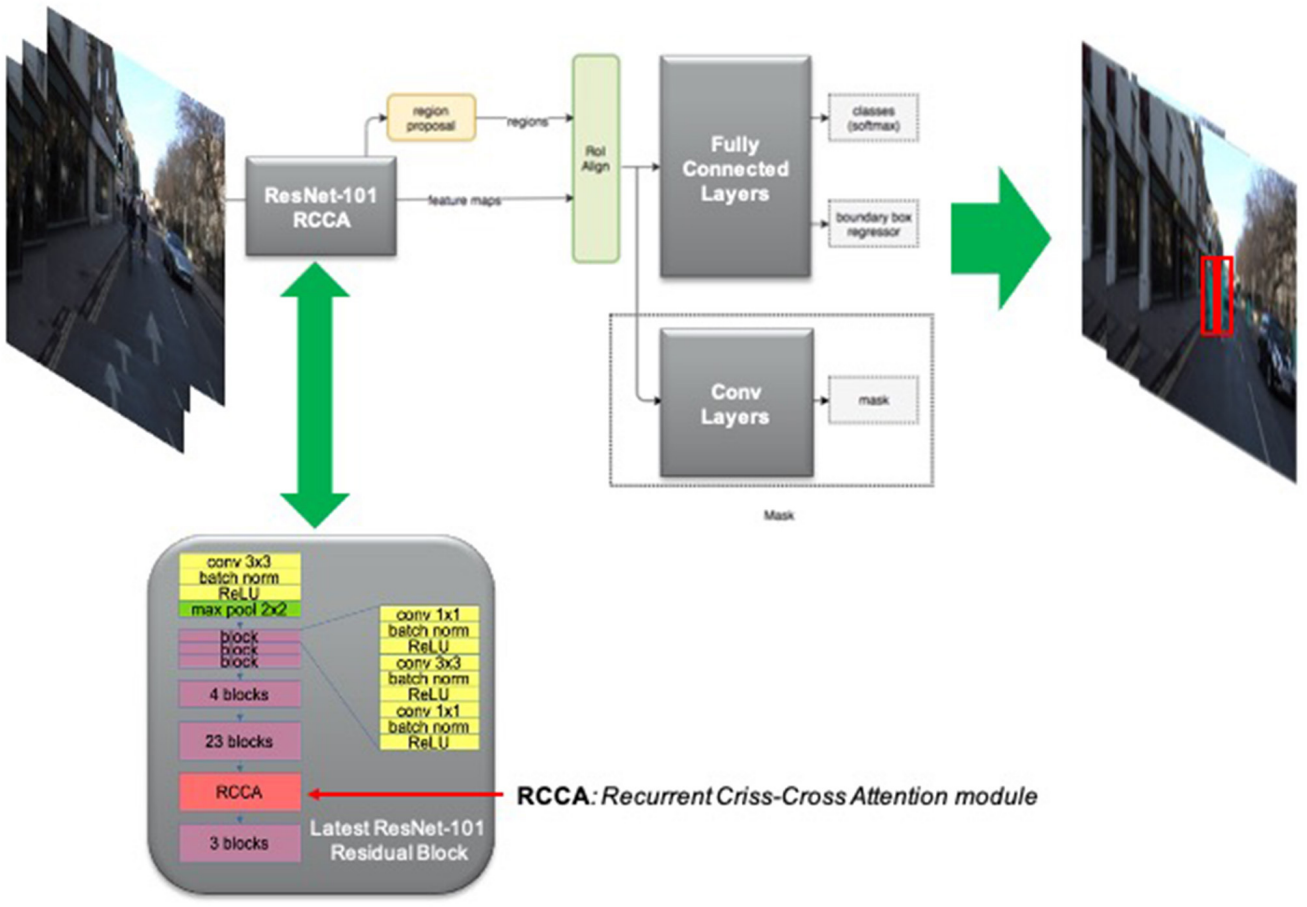
The proposed Pedestrian Tracking System based on Criss-Cross enhanced Mask-R-CNN.

The proposed Criss-Cross layer has been embedded in the Mask-R-CNN architecture used as pedestrian detection, segmentation and tracking. The architecture of Mask-R-CNN has been descripted in He et al. (2017). The Mask-R-CNN architecture extends previous detection and segmentation similar solutions such as Fast-R-CNN or Faster R-CNN by adding a branch-pipeline for predicting an object mask in parallel with the existing pipeline for bounding box recognition (He et al., 2017). One of the main parts of the Mask-R-CNN architecture is the Deep convolutional network used for the feature maps extraction. For this purpose, different backbones have been tested in the scientific literature (He et al., 2017, 2020). We denote the used backbone architecture using the nomenclature *network-depth-features* (He et al., 2017, 2020). In this proposed work, we used the Mask-R-CNN with a backbone based on a 2D ResNet-101 (He et al., 2017, 2020) in which we embedded, in the last block, a layer of self attention based on the aforementioned Criss-Cross methodology. The following Figure 13 shows the overall scheme of the proposed Criss-Cross enhanced Mask-R-CNN and some instances of the detected and segmented pedestrians both with red bounding-boxes and without. This architecture performed very well as we reached a test-set performance mIoU of 0.695 over CamVid dataset which is in line with the performance of other more complex architectures (He et al., 2017, 2020). Obviously, it is reiterated that the target of the work herein described in this manuscript is not to propose an architecture that outperforms the others in relation to the detection, tracking and segmentation of pedestrians but rather a system that detects the level of driving safety in risky scenarios. This proposed enhanced Criss-Cross architecture has been used as it presents an excellent trade-off between segmentation performance and complexity for an embedded system (as mentioned, this pipeline is being ported over the STA1295 MCU system). Furthermore, the Mask-R-CNN also allows us to obtain the bounding-box of the pedestrian which we will need to determine the distance from the driver's car. Quite simply, the height and width of the segmentation bounding box of each segmented pedestrian will be determined. Only bounding-boxes that have at least one of the two dimensions greater than two heuristically fixed thresholds (*L*_1_ and *L*_2_, respectively for length and width) will be considered salient pedestrians, i.e., pedestrians that must be considered by the driver when choosing the driving dynamics. The other pedestrians will be considered non salient and therefore not involved in safety level assessment. This so computed distance assessment will be used in the next block of the proposed pipeline.

### 5.6. The Driving Safety Monitoring System

We proposed an innovative pipeline for monitoring the car driving safety by means of visual and physiological data analysis. Specifically, the designed pipeline is able to combine the driver's physiological and visual data sampled in *ad-hoc* implemented sensing framework embedded in the car. We developed a system that was non-invasive for the driver, addressing the classic critical issues based on the usage of such physiological bio-signal difficulty to sample in a vehicle (ECG or EEG) or which require such sensors to be worn by the driver. For these reasons we have implemented a solution based on the usage of less-invasive car driver's PPG signal processing. When that PPG signal is no longer available for some reason, a parallel pipeline based on the usage of car driver visual data will be able to reconstruct specific features of the missed driver's PPG signal. From these estimated features we can reconstruct the level of driving safety by monitoring the driver's fatigue level i.e., a degree of attention through the analysis of Heart Rate Variability (HRV) and tracing the correlated blood pressure dynamics. Moreover, in order to increase the robustness of the proposed approach, a further visual driver face processing has been implemented trough *ad-hoc* D-CNN which will provide a classification of the car driver attention.

**Figure 14:**
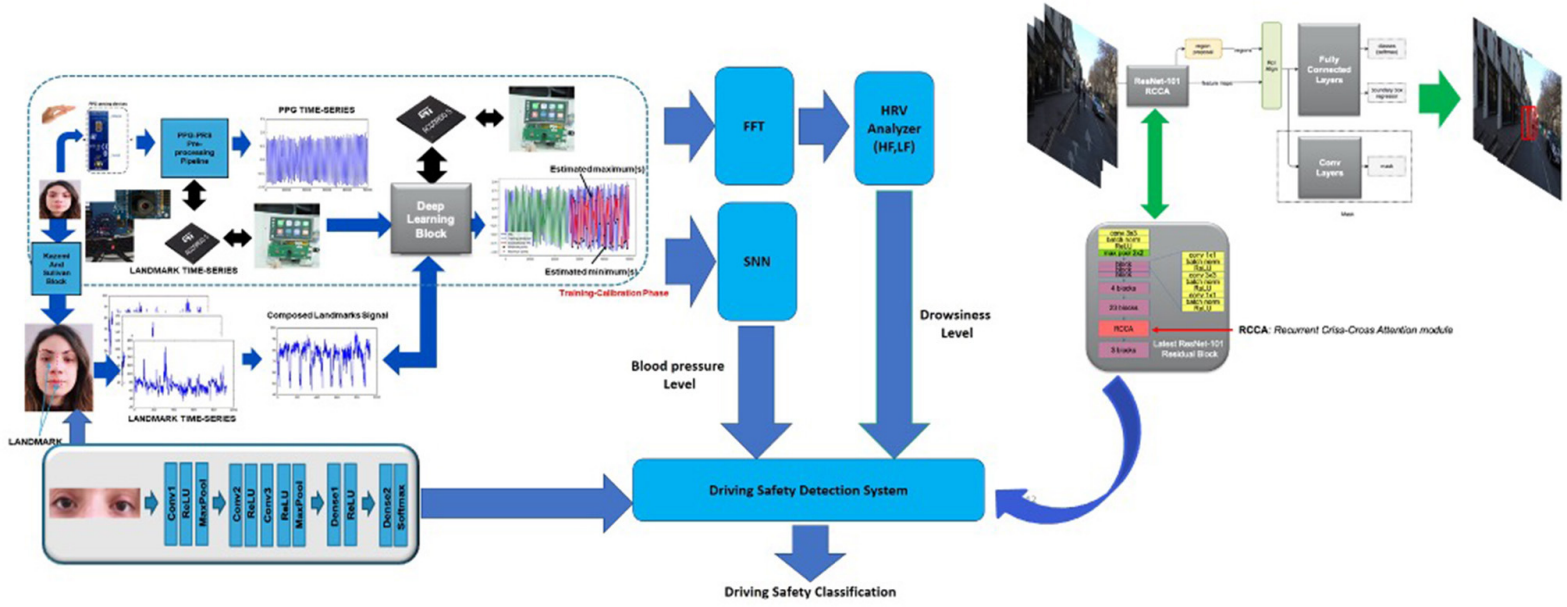
The overall scheme of the proposed solution with a detail of the designed Driving Safety Detection System.

The proposed application use-case is mainly aimed at driving in risky conditions, for instance, in scenarios in which one or more pedestrians are moving in the driving scene. By means of a modified Mask-R-CNN network with an attention layer through Criss-Cross methodology, we are able to detect and segment pedestrians in different configurations. Furthermore, we are able to obtain bounding-box segmentation and tracking which will allow us to estimate the distance of the pedestrian from the car. A comprehensive scheme of the proposed driving safety estimation architecture is showed in Figure 14. In this scheme is highlighted a block called Driving Safety Detection System (DSDS) which will process the outputs obtained by each of the previously described pipelines, specifically, the assessment of the driver's attention level (HRV analysis), the driver's blood pressure level (SNN output), the classification of the driver attention level performed by the implemented D-CNN and the distance of the detected and segmented pedestrians from the car. In detail, the DSDS will trigger an acoustic alert-signal, with different intensity according to the risk level, if one of the following setup will come true:

- High Risk Level (Alert-Signal with High intensity) HRV shows low attention level *AND* (the SNN output shows abnormal blood pressure *OR* D-CNN shows low attention level) *AND* the Mask-R-CNN identifies such salient pedestrians;
- Medium-Low Risk Level (Alert-Signal with Medium-Low intensity) HRV shows low attention level *OR* D-CNN shows low attention level *AND* the Mask-R-CNN identifies such salient pedestrians;

The acoustic signal is managed by the STA1295A Accordo5 Audio sub-system which will host the DSDS software implementation (see Figure 12). The system will therefore provide an assessment of the driving safety in the analyzed use-case: pedestrian tracking. All the developed sub-systems of the proposed pipeline are ongoing to be ported over the STA1295 Accordo5 Dual ARM Cortex MCUs platform.

### 5.7. Dataset

Under the supervision of physiologists, a dataset of selected and monitored subjects was collected. More in details, for each recruited subject we performed an acquisition of the PPG signal simultaneously with systolic and diastolic blood pressure measurements and contextually with a session of face video-recording. All data collections were conducted by inducing in both subjects such states of high attention and states of low attention that the physiologists who supervised the clinical study properly induced. More in details, for each recruited subject the EGG signal was sampled as well as the EEG time-series from which the physiologist analyze the dynamic: alpha waves representative of low attention status or beta waves representative of medium-high attention level (Guo and Markoni, 2019; Rundo et al., 2019b). Participants were recruited after signing the informed consent form provided by the Ethical Committee CT1 (authorization n.113/2018/PO). The dataset (further recruitments with respect to the work described in Rundo et al., 2018b, 2019b) is composed of 71 subjects (males and females, min age: 20 years, max age: 75 years) splitted into 45 subjects having a less or equal to 120/80 mmHg and 26 subjects having a blood pressure higher than 120/80 mmHg both in physiological and pathological state.

To carry out our experiments, we paid attention to the subjects with an arterial pressure greater or lower than 15% in average with respect to normal values (configured during the training-calibration phase of the Vision2PPG reconstruction pipeline for each subject). In case of hypotensive or hypertensive subjects, we have adjusted the reference blood pressure accordingly. The overall study was carried out in accordance with the protocol of the Declaration of Helsinki. For each subject, the blood pressure measurements were certified by means of a medical sphygmomanometer device. The PPG was sampled by the proposed sensing device at 1 kHz frequency. For retrieving visual data, we have used a commercial color camera device having max resolution of 2.3 Mpx and 50 fps as frame-rate. The collected minimum pressure value is around 98/70, while the maximum pressure value is 158/90. Each measurement session lasts 10 min, 5 of which in a state of high attention and 5 in a state of low attention. The level of adequate attention has been induced by performing mathematical calculations, reading anecdotes or by viewing representative driving scenarios where high driver attention is required (car overtaking, changing lane, braking, etc.). During this phase the subjects' EEG signal was sampled, confirming the presence of beta waves (Rundo et al., 2019b). Similarly, for low attention measurement sessions in which subjects were asked to relax, close their eyes for a few moments, listen slow music that induces states of relaxation, thus inducing a decrease in heart rate and the simultaneous presence of typical visual expressions showing drop in attention such as the decrease in the frequency of eye blinking. Only when the EEG showed the dynamic of alpha waves, the data (PPG and visual) were acquired so as to have certainty of the low attention state of the analyzed subject. Same approach for high attention states corresponding to beta waves. We divided the dataset as follows: 70% of the data has been used for the training while the remaining 30% for validation (15%) and testing (15%). We have run our experiments as well as training and testing of the proposed deep learning architectures in MATLAB full toolboxes rel. 2019b environment running in a server having an Intel 16-Cores and NVIDIA GeForce RTX 2080 GPU.

## 6. Results

In order to validate the proposed composed approach we have tested each of the proposed blocks i.e., the PPG-based HRV driver drowsiness detection as well as the driver blood pressure level estimation and finally the visual drowsiness estimation based on D-CNN processing and the pedestrian detection, segmentation and tracking. About the proposed PPG based HRV based drowsiness detection pipeline, the following Table 1 reports the overall testing accuracy of the approach as well as accuracy for each classified class.

**Table 1:** Car driver HRV drowsiness monitoring performance.

**Method**	**Overall accuracy (%)**	**Class 1 drowsy driver accuracy (%)**	**Class 2 driver accuracy (%)**
Proposed LSTM	95.07	95.77	94.36
Proposed 1D-TDCNN	95.67	96.33	94.78
Trenta et al. (2019) with LSTM	94.43	95.10	93.77
Rundo et al. (2019c)	93.66	95.77	91.54
MLP	87.94	87.55	88.33
SVM	88.05	86.98	89.11
ResNet-50	93.90	93.85	93.95

A description of the hardware and software system implemented to obtain these performances is reported. Four PPG sensing probes have been embedded in the car steering with the characteristics described in the introductory section of this paper having near infrared emission LEDs at 850 nm. In addition, a camera with characteristics of 50 fps and a maximum resolution of 2.3 Mpx has been mounted in the base of the steering wheel and directed to sample the driver's face. As described in the Vision2PPG reconstruction section, the vision system has been calibrated to reconstruct the PPG features processed as per Equations (9)–(13). The driver's PPG signal data sampled by the sensing probes will be then converted by the analog-to-digital converters (ADCs) embedded in the SPC5x Chorus MCU used to acquire and pre-process the raw PPG signal from the sensors. Furthermore, the so collected raw PPG signal will be stabilized and filtered in the range 0.5–10 Hz by means of the previously introduced PPG-PRS pipeline running as firmware in the mentioned embedded MCU. The visual frames of the Vision2PPG block will instead be stored in the DRAM of the embedded system based on STA1295A Accordo5 MCU in which the landmarks detection system based on the Kazemi and Sullivan approach is running. Therefore, the results reported in Table 1 were obtained by creating an application use-case lasting 45 minutes of acquisition of different scenarios, specifically ones in which the PPG signal was available (the driver placed the hand over the PPG probes embedding on the steering) and another ones in which this PPG signal was no longer available and therefore the Vision2PPG reconstruction block start the reconstruction of the PPG features. The PPG signal is sampled at a frequency of 1 Khz. The used ADCs embedded in the SPC5x CHORUS have a resolution of 12 bits. The set of PPG features have been reconstructed by Vision2PPG block through the usage of both D-LSTM (three layers of 64,64,128 cells trained with an initial learning rate of 10-3, a batch size set to 512 and with the maximum number of training epochs set to 200) and 1D-TCNN architectures (25 blocks -convolutions, normalization layer, spatial dropout, ReLU, residual block-, kernel 3 × 3, dilated convolutional with factor starting from 2 to 16, mini Batch Size of 1; initial learning rate equal to 0.001; and a Dropout Factor rated of 0.1). A comparison with similar approaches based on different methodologies have been reported in Table 1. More specifically, we have compared our architecture with our previous solution described in Rundo et al. (2019c) and Trenta et al. (2019) as well with an approach based on the usage of Support Vector Machine (SVM), Multi-layer Perceptron (MLP having an hidden layer of 600 neurons and trained with a more performer Levenberg-Marquardt algorithm) and finally with a ResNet-50, arranging the input landmarks in a 224 × 224 matrix. As highlighted from the reported benchmark comparison results, the method we propose and based on the use of deep 1D-TCNN network outperforms the others not only in terms of accuracy but rather in robustness as can also be seen from Table 2. Specifically, the method we propose presents a noteworthy advantages in terms of robustness compared to the previous version (Trenta et al., 2019) in real scenarios in which the driver wears sunglasses or eyeglasses (highly probable scenarios). Furthermore, in terms of computational complexity and therefore implementation costs, our solution based on 1D-TDCNN networks shows a lower complexity than the architectures that show comparable performances: ResNet-50 has a size of 98 Mb against the 42 Mb of our 1D-TDCNN. This aspect is very important considering that this solution must be applied in the automotive field over the embedded platforms mentioned in the previous sections, confirming an undoubted advantage in terms of sustainability costs with greater performance in terms of accuracy. The accuracy reported in Table 1 was obtained by analyzing the HRV associated with the features of the PPG signal thus processed, by analyzing the HF, LF, and HF/LF ratio indicators (see section 5.3.1). We remark that most significant advantage of the proposed method is non-invasiveness in that it does not require the driver to wear any sensor nor does it requires the driver necessarily places the hand on the part of the steering in which the PPG sensors are housed. Similarly, in the same dataset, we validated the reconstruction of the driver's blood pressure level through the SNN network. For this testing session, we used the same aforementioned hardware setup of the PPG sensing probes. The collected PPG features (both native from sensors and reconstructed from Vision2PPG block) will be fed to the implemented SNN Fully Connected Multi-Layers Network having 64 neurons as input layer joined with two hidden layers of 500 and 300 neurons and a binary output. The network was trained with Scaled Conjugate Gradient backpropagation (SCG) algorithm and compared with other methodologies such as MLP, SVM, ResNet-50, and previous method described in Trenta et al. (2019). The validation scenario consists in constantly monitoring the subject's blood pressure level (by means of a digital blood pressure detection device embedded in the driver's arm) and detecting the classification reconstructed by our SNN output system (output in the range 0–0.5 means pressure within the norm while values above 0.5 indicate abnormal blood pressure of the driver). Once again, an examination of the benchmark comparison values reported in Table 3 confirms the evident advantages of the proposed pipeline as it not only outperforms in accuracy our previous solution reported in Rundo et al. (2019a) and the other methodologies tested but, in terms of complexity is significantly lower than a solution based on deep learning (ResNet-50). Furthermore, the pipeline herein described allows to overcome the raised limit of the previous version reported in Trenta et al. (2019) which had no significant accuracy in the classification of the subject's blood pressure level.

**Table 2:** Car driver's face Landmarks detection methods: Robustness comparison.

**Method**	**Overall accuracy car driver NS (%)**	**Overall accuracy car driver WS (%)**	**Class 2 accuracy car Driver WE (%)**
Trenta et al. (2019) with LSTM	94.43	0.00	70.16
Proposed 1D-TCNN	95.67	92.31	93.98

*(NS, normal scenario; WS, wearing sunglasses; WE, wearing eyeglasses)*.

**Table 3:** Blood pressure (BP) performance.

**Method**	**Overall accuracy (%)**	**Class 1 normal BP accuracy (%)**	**Class 2 abnormal BP accuracy (%)**
Proposed	90.14	88.88	92.30
Rundo et al. (2019a)	88.73	91.11	84.61
Trenta et al. (2019) with LSTM	<50	<50	<50
MLP	89.47	87.62	91.33
SVM	82.24	81.98	82.05
ResNet-50	90,01	89.02	91.01

**Figure 15:**
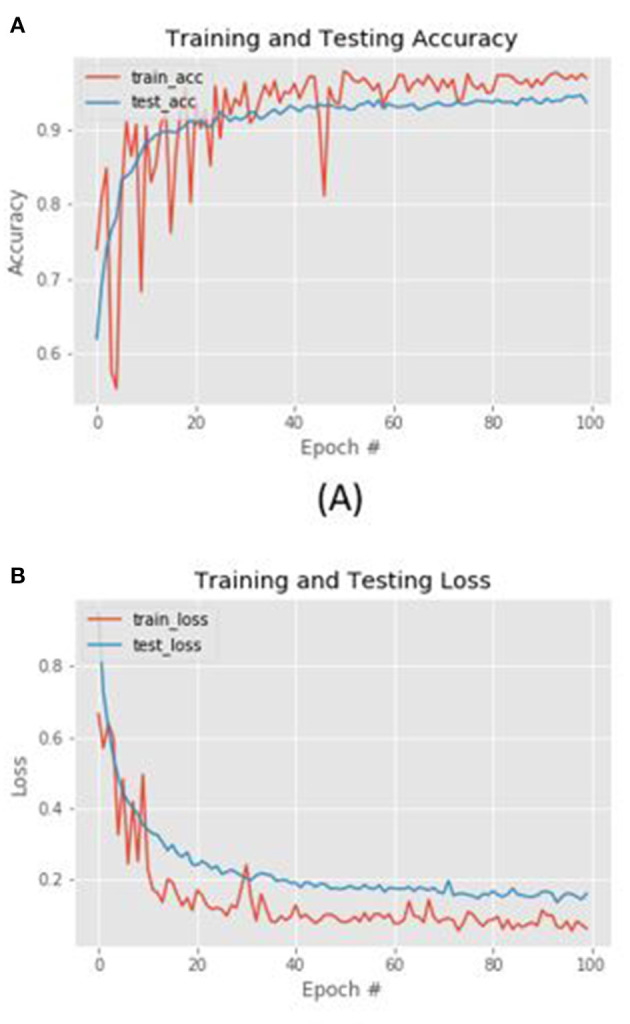
For the proposed D-CNN performance diagrams: **(A)** Training and testing accuracy **(B)** training and testing loss.

A comparison with another approach (Rundo et al., 2019c) is reported. As evident from comparison data reported in Table 1, the proposed method performs very well (both by using the D-LSTM and 1D-TDCNN backbone) in that it shows overall accuracy and accuracy for each classified class slightly greater to the compared methods. The significant advantage of the proposed method is non-invasiveness in that it does not require the driver to wear any sensor nor does it requires the driver necessarily places his/her hand on the part of the steering in which the PPG signal sensors are housed. Similarly, in the same dataset, we validated the reconstruction of the driver's blood pressure level through the SNN network trained with the features extracted from PPG signal (or from the Vision2PPG reconstruction block). Also, in this case we have compared our method with others reported in the literature (Rundo et al., 2019a). From Table 3, the performance of the proposed method is very promising as we are able to classify normal-pressure subjects (with an accuracy of 88.88% with respect to 91.11% showed by similar PPG based pipeline; Rundo et al., 2019a) from those who instead have a pressure level out of range (accuracy of 92.30%) with an overall accuracy of 90.14% relatively outperforming with respect to the similar methods based on PPG signal sampling. We tested the D-CNN based pipeline for tracking and classifying the visual features extracted from the driver's face during the measurement sessions. Also in this case we have obtained an accuracy about of 90% in the testing dataset confirming the robustness of the proposed approach. In Figure 15, we show the loss and accuracy related to the D-CNN learning and testing phase. Although the target of this proposal is not the detection, tracking and segmentation of pedestrians in the driving scene, we have still validated the deep architecture we have developed by including a Self Attention layer based on Criss-Cross processing in the Mask-R-CNN network with ResNet-101 as the backbone. As explained, that enhanced Mask-R-CNN was used as simple application use-case for checking driving safety by mean of the proposed pipeline. As introduced in the previous section, we obtained in the testing phase a performance mIoU of 0.695 over CamVid dataset which outperforms other similarly complex architectures described in the literature (He et al., 2017, 2020).

## 7. Conclusions and Discussion

In this study, we addressed the mentioned issue regarding the estimation of driving safety by using a non-invasive and robust methodology. Contrary to existing approaches, our proposed method does not require contact with the driver or the necessity to wear PPG sensors to collect the physiological signal. The advantages of non-invasiveness are however accompanied by overlapping (and sometimes even higher) performances than those obtained by the classic methods which, however, require the use of invasive sensing devices. The proposed multi-modal approach that involves the use of visual and physiological data and to correlate to each other (by means of Vision2PPG reconstruction system) allows to obtain high fault tolerance performance. The experimental results allow to be confident about the applicability of the proposed Vison2Physio approach in different scientific applications. The implemented pipeline is ongoing to be ported to SPC5 Chorus based platform (PPG sensing and processing) while the deep learning architectures will be ported in the STA1295A Accordo5 embedded automotive platform in which two ARM Cortex A7 and 3D GFX accelerator hardware are able to host the developed software as firmware. The embedded Operating System used for both applications is *ad-hoc* YOCTO Linux distribution released for this specific kind of application. The use-case analyzed in this proposal concerns the intelligent tracking of pedestrians in a safe driving scenario. However, the implemented approach can be successfully used in several other automotive use cases. For instance, we have implemented a safe driving application in which, through a deep architecture based on Fully Convolutional Neural Network with Self Attention, we are able to classify the level of risk of the driving scenario and at the same time the driver drowsiness through the PPG-based monitoring pipeline herein described (Rundo et al., 2020a). Another automotive issue we have addressed is the robust identification of the car driver. Through *ad-hoc* intelligent pipeline based on the usage of PPG signal and deep network, we have designed the so called “physiological fingerprint” of the driver used for an effective identification (Rundo et al., 2020d). Therefore the proposed method can be generalized and applied in various automotive scenarios in which it is necessary to characterize the level of attention of the driver or in all the use-cases that deal with driving safety. As future development, we plan to collect more data with the aim of improving the effectiveness of the proposed approach. Specifically, we will address further application in the automotive field with special focus to autonomous driving and ADAS systems during low-light driving scenarios both inside and outside the car.

## Data Availability Statement

The datasets presented in this article are not readily available because of confidentiality restrictions related to registered patents. Requests to access the datasets should be directed to francesco.rundo@st.com.

## Ethics Statement

The studies involving human participants were reviewed and approved by Ethical Committee CT1 (authorization n.113/2018/PO). The patients/participants provided their written informed consent to participate in this study. Written informed consent was obtained from the individual(s) for the publication of any potentially identifiable images or data included in this article.

## Author Contributions

FR and FT worked on data collection and data analysis. FR, RL, FT, and CS worked on writing of the manuscript. SC and SB supervised the research and worked on data collection. All authors contributed to the article and approved the submitted version.

## Conflict of Interest

FR was employed by the company STMicroelectronics. The remaining authors declare that the research was conducted in the absence of any commercial or financial relationships that could be construed as a potential conflict of interest.

## Publisher's Note

All claims expressed in this article are solely those of the authors and do not necessarily represent those of their affiliated organizations, or those of the publisher, the editors and the reviewers. Any product that may be evaluated in this article, or claim that may be made by its manufacturer, is not guaranteed or endorsed by the publisher.

## Footnotes

^1^ https://www.who.int/gho/ncd/risk_factors/blood_pressure_prevalence_text/en/

^2^ https://ec.europa.eu/transport/road_safety/sites/roadsafety/files/ersosynthesis2015-fatigue25_en.pdf

^3^ https://www.st.com/en/automotive-infotainment-and-telematics/sta1295.html

## References

[B1] Agró D. Canicattí R. Tomasino A. Giordano A. Adamo G. Parisi A.. (2014). PPG embedded system for blood pressure monitoring, in 2014 AEIT Annual Conference-From Research to Industry: The Need for a More Effective Technology Transfer (AEIT), 1–6. 10.1109/AEIT.2014.7002012

[B2] Alty S. R. Angarita-Jaimes N. Millasseau S. C. Chowienczyk P. J. (2007). Predicting arterial stiffness from the digital volume pulse waveform. IEEE Trans. Biomed. Eng. 54, 2268–2275. 10.1109/TBME.2007.897805 18075043

[B3] Balakrishnan G. Durand F. Guttag J. (2013). Detecting pulse from head motions in video, in Proceedings of the IEEE Conference on Computer Vision and Pattern Recognition, 3430–3437. 10.1109/CVPR.2013.440

[B4] Battiato S. Conoci S. Leotta R. Ortis A. Rundo F. Trenta F. (2020). Benchmarking of computer vision algorithms for driver monitoring on automotive-grade devices, in 2020 AEIT International Conference of Electrical and Electronic Technologies for Automotive (AEIT AUTOMOTIVE), 1–6. 10.23919/AEITAUTOMOTIVE50086.2020.9307437

[B5] Bhola G. Kathuria A. Kumar D. Das C. (2020). Real-time pedestrian tracking based on deep features, in 2020 4th International Conference on Intelligent Computing and Control Systems (ICICCS), 1101–1106. 10.1109/ICICCS48265.2020.9121061

[B6] Choi M. Koo G. Seo M. Kim S. W. (2017). Wearable device-based system to monitor a driver's stress, fatigue, and drowsiness. IEEE Trans. Instrument. Measure. 67, 634–645. 10.1109/TIM.2017.2779329

[B7] Choi Y. Han S. I. Kong S.-H. Ko H. (2016). Driver status monitoring systems for smart vehicles using physiological sensors: a safety enhancement system from automobile manufacturers. IEEE Signal Process. Mag. 33, 22–34. 10.1109/MSP.2016.2602095

[B8] Conoci S. Rundo F. Fallica G. Lena D. Buraioli I. Demarchi D. (2018). Live demonstration of portable systems based on silicon sensors for the monitoring of physiological parameters of driver drowsiness and pulse wave velocity, in 2018 IEEE Biomedical Circuits and Systems Conference (BioCAS), 1–3. 10.1109/BIOCAS.2018.858470929780971

[B9] Dastjerdi A. E. Kachuee M. Shabany M. (2017). Non-invasive blood pressure estimation using phonocardiogram, in 2017 IEEE International Symposium on Circuits and Systems (ISCAS), 1–4. 10.1109/ISCAS.2017.8050240

[B10] Deng W. Wu R. (2019). Real-time driver-drowsiness detection system using facial features. IEEE Access 7, 118727–118738. 10.1109/ACCESS.2019.2936663

[B11] Guo J.-M. Markoni H. (2019). Driver drowsiness detection using hybrid convolutional neural network and long short-term memory. Multimed. Tools Appl. 78, 29059–29087. 10.1007/s11042-018-6378-6

[B12] He K. Gkioxari G. Dollár P. Girshick R. (2017). Mask R-CNN, in Proceedings of the IEEE International Conference on Computer Vision, 2961–2969. 10.1109/ICCV.2017.322

[B13] He K. Gkioxari G. Dollar P. Girshick R. (2020). Mask r-cnn. IEEE Trans. Pattern Anal. Mach. Intell. 42, 386–397. 10.1109/TPAMI.2018.2844175 29994331

[B14] Hochreiter S. Schmidhuber J. (1997). Long short-term memory. Neural Comput. 9, 1735–1780. 10.1162/neco.1997.9.8.1735 9377276

[B15] Huang Z. Wang X. Huang L. Huang C. Wei Y. Liu W. (2019). CCNET: Criss-cross attention for semantic segmentation, in Proceedings of the IEEE/CVF International Conference on Computer Vision, 603–612. 10.1109/ICCV.2019.0006932750802

[B16] Hui X. Kan E. C. (2019). Seat integration of RF vital-sign monitoring, in 2019 IEEE MTT-S International Microwave Biomedical Conference (IMBioC), Vol. 1, 1–3. 10.1109/IMBIOC.2019.8777742

[B17] Husodo A. Y. Hermawan I. Jatmiko W. Wiweko B. Boediman A. Pradekso B. K. (2018). Multi-parameter measurement tool of heart rate and blood pressure to detect Indonesian car drivers drowsiness, in 2018 3rd International Seminar on Sensors, Instrumentation, Measurement and Metrology (ISSIMM), 68–73. 10.1109/ISSIMM.2018.8727729

[B18] Huynh T. H. Jafari R. Chung W.-Y. (2018). Noninvasive cuffless blood pressure estimation using pulse transit time and impedance plethysmography. IEEE Trans. Biomed. Eng. 66, 967–976. 10.1109/TBME.2018.2865751 30130167

[B19] Jabbar R. Shinoy M. Kharbeche M. Al-Khalifa K. Krichen M. Barkaoui K. (2020). Driver drowsiness detection model using convolutional neural networks techniques for android application, in 2020 IEEE International Conference on Informatics, IoT, and Enabling Technologies (ICIoT), 237–242. 10.1109/ICIoT48696.2020.9089484

[B20] Jeon H.-M. Nguyen V. D. Jeon J. W. (2019). Pedestrian detection based on deep learning, in IECON 2019-45th Annual Conference of the IEEE Industrial Electronics Society, Vol. 1, 144–151. 10.1109/IECON.2019.8927417

[B21] Kazemi V. Sullivan J. (2014). One millisecond face alignment with an ensemble of regression trees, in Proceedings of the IEEE Conference on Computer Vision and Pattern Recognition, 1867–1874. 10.1109/CVPR.2014.241

[B22] Koh S. Cho B. R. Lee J.-I. Kwon S.-O. Lee S. Lim J. B.. (2017). Driver drowsiness detection via ppg biosignals by using multimodal head support, in 2017 4th International Conference on Control, Decision and Information Technologies (CoDIT), 383–388. 10.1109/CoDIT.2017.8102622

[B23] Kurian D. PL J. J. Radhakrishnan K. Balakrishnan A. A. (2014). Drowsiness detection using photoplethysmography signal, in 2014 Fourth International Conference on Advances in Computing and Communications, 73–76. 10.1109/ICACC.2014.23

[B24] Lee B.-G. Jung S.-J. Chung W.-Y. (2011). Real-time physiological and vision monitoring of vehicle driver for non-intrusive drowsiness detection. IET commun. 5, 2461–2469. 10.1049/iet-com.2010.0925

[B25] Lee H. Lee J. Shin M. (2019). Using wearable ECG/PPG sensors for driver drowsiness detection based on distinguishable pattern of recurrence plots. Electronics 8:192. 10.3390/electronics8020192

[B26] Littler W. A. Honour A. Sleight P. (1973). Direct arterial pressure and electrocardiogram during motor car driving. Br. Med. J. 2, 273–277. 10.1136/bmj.2.5861.273 4704496 PMC1589169

[B27] Liu J. Yan B. P.-Y. Dai W.-X. Ding X.-R. Zhang Y.-T. Zhao N. (2016). Multi-wavelength photoplethysmography method for skin arterial pulse extraction. Biomed. Opt. Exp. 7, 4313–4326. 10.1364/BOE.7.004313 27867733 PMC5102532

[B28] Mazzillo M. Condorelli G. Sanfilippo D. Valvo G. Carbone B. Fallica G.. (2009). Silicon photomultiplier technology at stmicroelectronics. IEEE Trans. Nucl. Sci. 56, 2434–2442. 10.1109/TNS.2009.2024418

[B29] Mazzillo M. Maddiona L. Rundo F. Sciuto A. Libertino S. Lombardo S.. (2018). Characterization of SiPMs with NIR long-pass interferential and plastic filters. IEEE Photon. J. 10, 1–12. 10.1109/JPHOT.2018.2834738

[B30] Monte-Moreno E. (2011). Non-invasive estimate of blood glucose and blood pressure from a photoplethysmograph by means of machine learning techniques. Artif. Intell. Med. 53, 127–138. 10.1016/j.artmed.2011.05.001 21696930

[B31] Oh T.-H. Jaroensri R. Kim C. Elgharib M. Durand F. Freeman W. T.. (2018). Learning-based video motion magnification, in Proceedings of the European Conference on Computer Vision (ECCV), 633–648. 10.1007/978-3-030-01225-0_39

[B32] Rubinstein M. Wadhwa N. Durand F. Freeman W. T. Wu H.-Y. (2013). Revealing invisible changes in the world. Science 339:519.

[B33] Rundo F. Petralia S. Fallica G. Conoci S. (2019d). A nonlinear pattern recognition pipeline for PPG/ECG medical assessments, in Sensors. CNS 2018. Lecture Notes in Electrical Engineering, Vol. 539, eds Andò B.. (Cham: Springer). 10.1007/978-3-030-04324-7_57

[B34] Rundo F. Conoci S. Battiato S. Trenta F. Spampinato C. (2020a). Innovative saliency based deep driving scene understanding system for automatic safety assessment in next-generation cars, in 2020 AEIT International Conference of Electrical and Electronic Technologies for Automotive (AEIT AUTOMOTIVE), 1–6. 10.23919/AEITAUTOMOTIVE50086.2020.9307425

[B35] Rundo F. Conoci S. Fallica P. G. (2021). Method of Processing Electrophysiological Signals and Corresponding System, Vehicle, and Computer Program Product. USA Patent Nr. 10987007.

[B36] Rundo F. Conoci S. Ortis A. Battiato S. (2018a). An advanced bio-inspired photoplethysmography (PPG) and ECG pattern recognition system for medical assessment. Sensors 18:405. 10.3390/s18020405 29385774 PMC5855408

[B37] Rundo F. Ortis A. Battiato S. Conoci S. (2018b). Advanced bio-inspired system for noninvasive cuff-less blood pressure estimation from physiological signal analysis. Computation 6:46. 10.3390/computation6030046

[B38] Rundo F. Ortis A. Battiato S. Conoci S. (2019a). Advanced multi-neural system for cuff-less blood pressure estimation through nonlinear HC-features, in ICETE (1), 327–331. 10.5220/0007909403210325

[B39] Rundo F. Rinella S. Massimino S. Coco M. Fallica G. Parenti R.. (2019b). An innovative deep learning algorithm for drowsiness detection from EEG signal. Computation 7:13. 10.3390/computation7010013

[B40] Rundo F. Spampinato C. Battiato S. Trenta F. Conoci S. (2020b). Advanced 1d temporal deep dilated convolutional embedded perceptual system for fast car-driver drowsiness monitoring, in 2020 AEIT International Conference of Electrical and Electronic Technologies for Automotive (AEIT AUTOMOTIVE), 1–6. 10.23919/AEITAUTOMOTIVE50086.2020.9307400

[B41] Rundo F. Spampinato C. Conoci S. (2019c). *Ad-hoc* shallow neural network to learn hyper filtered photoplethysmographic (PPG) signal for efficient car-driver drowsiness monitoring. Electronics 8:890. 10.3390/electronics8080890

[B42] Rundo F. Spampinato C. Conoci S. Trenta F. Battiato S. (2020c). Deep bio-sensing embedded system for a robust car-driving safety assessment, in 2020 AEIT International Conference of Electrical and Electronic Technologies for Automotive (AEIT AUTOMOTIVE), 1–6. 10.23919/AEITAUTOMOTIVE50086.2020.9307409

[B43] Rundo F. Trenta F. Leotta R. Spampinato C. Piuri V. Conoci S.. (2020d). Advanced temporal dilated convolutional neural network for a robust car driver identification, in ICPR Workshops, 184–199.

[B44] Schmidt Robert F. Thews G. (1989). Autonomic nervous system, in Human Physiology, 2nd Edn, ed Janig W., 333–370. 10.1007/978-3-642-73831-9_16

[B45] Slapničar G. Mlakar N. Luštrek M. (2019). Blood pressure estimation from photoplethysmogram using a spectro-temporal deep neural network. Sensors 19:3420. 10.3390/s19153420 31382703 PMC6696196

[B46] Song H. Choi I. K. Ko M. S. Bae J. Kwak S. Yoo J. (2018). Vulnerable pedestrian detection and tracking using deep learning, in 2018 International Conference on Electronics, Information, and Communication (ICEIC) (Honolulu, HI), 1–2. 10.23919/ELINFOCOM.2018.8330547

[B47] Tian Y. Luo P. Wang X. Tang X. (2015). Deep learning strong parts for pedestrian detection, in Proceedings of the IEEE International Conference on Computer Vision (Santiago), 1904–1912. 10.1109/ICCV.2015.221

[B48] Trenta F. Conoci S. Rundo F. Battiato S. (2019). Advanced motion-tracking system with multi-layers deep learning framework for innovative car-driver drowsiness monitoring, in 2019 14th IEEE International Conference on Automatic Face & Gesture Recognition (FG 2019) (Lille), 1–5. 10.1109/FG.2019.8756566

[B49] Vavrinský E. Tvarožek V. Stopjaková V. Soláriková P. Brezina I. (2010). Monitoring of car driver physiological parameters, in The Eighth International Conference on Advanced Semiconductor Devices and Microsystems (Smolenice), 227–230. 10.1109/ASDAM.2010.5667021

[B50] Vinciguerra V. Ambra E. Maddiona L. Oliveri S. Romeo M. F. Mazzillo M.. (2017). Progresses towards a processing pipeline in photoplethysmogram (PPG) based on SiPMs, in 2017 European Conference on Circuit Theory and Design (ECCTD) (Catania), 1–5. 10.1109/ECCTD.2017.8093327

[B51] Vinciguerra V. Ambra E. Maddiona L. Romeo M. Mazzillo M. Rundo F.. (2019). PPG/ECG multisite combo system based on SiPM technology, in Sensors. CNS 2018. Lecture Notes in Electrical Engineering, Vol. 539, eds Andò B.. (Cham: Springer). 10.1007/978-3-030-04324-7_44

[B52] Viola P. Jones M. (2001). Rapid object detection using a boosted cascade of simple features, in Proceedings of the 2001 IEEE Computer Society Conference on Computer Vision and Pattern Recognition, CVPR 2001, Vol. 1 (Kauai, HI). 10.1109/CVPR.2001.990517

[B53] Vural E. Cetin M. Ercil A. Littlewort G. Bartlett M. Movellan J. (2007). Drowsy driver detection through facial movement analysis, in International Workshop on Human-Computer Interaction (Rio de Janeiro: Springer), 6–18. 10.1007/978-3-540-75773-3_2

[B54] Wu C.-Y. Hu H.-Y. Chou Y.-J. Huang N. Chou Y.-C. Li C.-P. (2015). High blood pressure and all-cause and cardiovascular disease mortalities in community-dwelling older adults. Medicine 94:47. 10.1097/MD.0000000000002160 26632749 PMC5059018

[B55] Wu H.-Y. Rubinstein M. Shih E. Guttag J. Durand F. Freeman W. (2012). Eulerian video magnification for revealing subtle changes in the world. ACM Trans. Graph. 31, 1–8. 10.1145/2185520.2185561

[B56] Zhao W. Gao Y. Ji T. Wan X. Ye F. Bai G. (2019). Deep temporal convolutional networks for short-term traffic flow forecasting. IEEE Access 7, 114496–114507. 10.1109/ACCESS.2019.2935504

